# A Transcriptional Link between HER2, JAM-A and FOXA1 in Breast Cancer

**DOI:** 10.3390/cells11040735

**Published:** 2022-02-19

**Authors:** Rodrigo G. B. Cruz, Stephen F. Madden, Kieran Brennan, Ann M. Hopkins

**Affiliations:** 1 Department of Surgery, Royal College of Surgeons in Ireland, Beaumont Hospital, 9 Dublin, Ireland; rodrigogbc@hotmail.com (R.G.B.C.); ciaran.brennan2@gmail.com (K.B.); 2 Data Science Centre, Royal College of Surgeons in Ireland, 2 Dublin, Ireland; stephenmadden@rcsi.com

**Keywords:** breast cancer, HER2, JAM-A, FOXA1, survival, tight junction, transcription factor, bioinformatics, patients, gastric cancer

## Abstract

Overexpression of the human epidermal growth factor receptor-2 (HER2) is associated with aggressive disease in breast and certain other cancers. At a cellular level, the adhesion protein Junctional Adhesion Molecule-A (JAM-A) has been reported to regulate the expression of HER3 via a transcriptional pathway involving FOXA1. Since FOXA1 is also a suggested transcription factor for HER2, this study set out to determine if JAM-A regulates HER2 expression via a similar mechanism. An integrated tripartite approach was taken, involving cellular expression studies after targeted disruption of individual players in the putative pathway, in silico identification of relevant HER2 promoter regions and, finally, interrogation of cancer patient survival databases to deconstruct functionally important links between HER2, JAM-A and FOXA1 gene expression. The outcome of these investigations revealed a unidirectional pathway in which JAM-A expression transcriptionally regulates that of HER2 by influencing the binding of FOXA1 to a specific site in the HER2 gene promoter. Moreover, a correlation between JAM-A and HER2 gene expression was identified in 75% of a sample of 40 cancer types from The Cancer Genome Atlas, and coincident high mean mRNA expression of JAM-A, HER2 and FOXA1 was associated with poorer survival outcomes in HER2-positive (but not HER2-negative) patients with either breast or gastric tumors. These investigations provide the first evidence of a transcriptional pathway linking JAM-A, HER2 and FOXA1 in cancer settings, and support potential future pharmacological targeting of JAM-A as an upstream regulator of HER2.

## 1. Introduction

Breast cancers which overexpress the human epidermal growth factor receptor-2 (HER2) have been associated with aggressive clinical phenotypes including high grade tumors, increased growth rates, early metastasis, and decreased rates of both disease-free survival and overall survival [1,2]. It has been reported that high expression of the adhesion protein Junctional Adhesion Molecule-A (JAM-A) correlates with HER2 expression in breast cancer patient tissues, and that JAM-A regulates HER2 protein stability [3]. Initially, this seems at odds with the multiple physiological localizations [4,5] and functions (reviewed by [6]) of JAM-A, including intercellular tight junction assembly, cell polarity, leukocyte transmigration, platelet activation and angiogenesis. In the pathophysiological setting of cancer, however, JAM-A gene amplification or protein overexpression has recently emerged to positively correlate with aggressive disease and poor patient outcome in multiple carcinomas including breast [3,7,8], glioblastoma, nasopharyngeal, gastric and lung [9,10,11,12]. Furthermore, crosstalk between JAM-A and HER2 has been proposed as a novel contributor to the development of therapeutic resistance to HER2-targeted therapies in breast cancer settings [13].

Although the putative mechanism by which JAM-A regulates HER2 expression involves protection against proteasomal degradation of the latter [3], it is noteworthy that degradation of HER2 can take place either by lysosomal or proteasomal pathways [14]. Accordingly, we recently reported that lysosomal inhibition in breast cancer cells partially rescues HER2 protein loss downstream of JAM-A degradation induced by the antitumor antibiotic Tetrocarcin-A [15]. Thus, it is likely that the regulation of HER2 by JAM-A is complex and multifactorial, and may not be restricted to post-translational settings. Post-transcriptional regulation through microRNAs (miRs) could be a possibility. However, although several miRs that target HER2 mRNA for degradation have been identified (including miR-125a, miR-125b [16], miR-552, miR-541, miR-193a-5p, miR-453, miR-134, miR-498, and miR-331-3p [17]), none of these have been linked with JAM-A. There is also currently no known mechanism to explain why a membranous protein such as JAM-A might influence miRNA expression or function. Accordingly, there is also no mechanism to suggest why JAM-A levels would directly influence the stability of mRNA transcripts.

Taken together with recent evidence that JAM-A regulates expression of the HER2 family member HER3 by influencing the abundance or localization of FOXA1 and β-catenin transcription factors [18], the aim of this study was therefore to interrogate whether JAM-A could also influence HER2 expression at the earlier stage of transcriptional regulation. Since FOXA1 has been described not just as a transcription factor for HER3 [19,20] but also for HER2 [21], the current work focused on interrogating JAM-A/HER2 expressional links in the context of cancer patient survival, and as a starting point to address the cell biological pathways involved. Taken together, the results presented herein add to a growing body of evidence suggesting JAM-A as an upstream regulator of HER2-driven tumorigenic signaling and as a potential drug target that warrants future investigation.

## 2. Materials and Methods

### 2.1. Cell Culture

MCF7 and MDA-MB-231 cells were purchased from the American Tissue Culture Collection. SK-BR-3 cells were a kind gift from Dr. Alex Eustace and Dr. Norma O’Donovan (Dublin City University) [22]. Cells stably overexpressing JAM-A (termed MDA-MB-231-JAM+, MCF7-JAM+ and SK-BR-3-JAM+) were generated by stable transfection of full-length human JAM-A DNA into a pcDNA3 plasmid (kind gift from Prof. Charles Parkos, University of Michigan, USA) [23] into wild type cells and subsequent selection with G418. Cells stably overexpressing the pcDNA3 empty vector were used as controls, and termed simply as MDA-MB-231, MCF7 and SK-BR-3 in this work. MCF7-HER2 cells were a kind gift from Prof. Dennis Slamon (University College Los Angeles, via Dr. Norma O’Donovan, Dublin City University) [24,25].

MDA-MB-231 and MDA-MB-231-JAM+ cells were maintained in Dulbecco’s Modified Eagle Medium supplemented with 10% Fetal Bovine Serum, 50 U/mL penicillin, 50 μg/mL streptomycin and 2 mM L-glutamine. MCF7, MCF7-JAM+ and MCF7-HER2 cells were maintained in Eagle’s Minimum Essential Medium supplemented with 10% Fetal Bovine Serum, 50 U/mL penicillin, 50 µg/mL streptomycin, 2 mM L-glutamine and 1% non-essential amino acids. SK-BR-3 cells were maintained in RPMI-1640 medium supplemented with 10% Fetal Bovine Serum, 50 U/mL penicillin, 50 µg/mL streptomycin and 2 mM L-glutamine. They were maintained at 37 °C and 5% CO_2_ in a humidified environment and subcultured every 3–4 days in a sterile laminar flow hood using 1% trypsin in 0.02% EDTA solution, following aseptic technique. All cell lines were quarterly confirmed as mycoplasma-free using MycoAlert detection kits (Lonza, Basel, Switzerland). Cell lines were genotyped once yearly via Short Tandem Repeat sequencing to confirm their identity (Source BioScience, Nottingham, UK).

### 2.2. Gene Silencing

Short interfering RNA (siRNA) sequences targeting the genes of interest were transiently transfected into cells using Dharmafect-1 transfection reagent under antibiotic-free conditions. Specifically, cells were plated at 150,000 cells per well in 6-well plates 24 h prior to transfection and transfected the following day with 25 nM siRNA targeting the mRNA of the gene of interest. A non-targeting control siRNA pool (ON-TARGETplus Non-targeting Pool # D-001810-10-05, Dharmacon, Lafayette, IN, USA) consisting of a negative control pool of four siRNA sequences targeting no known human genes was used as a negative control. Cells were harvested after 72 h incubation at 37 °C with 5% CO_2_ (unless specified otherwise in individual figures). siRNA constructs were as follows:

JAM-A (F11R gene): NM_016946—SASI_Hs01_00049785, Sigma Aldrich (termed siJAM1) and a self-designed JAM-A siRNA manufactured by Dharmacon—Sense Sequence CGGGGGUCGCAGGAAUCUGUU (termed siJAM2). siJAM1 and siJAM2 were used either as a dual siRNA pool or individually (as specified in individual figure legends).

HER2: ON-TARGETplus ERBB2 siRNA SMARTpool (a mixture of 4 siRNAs provided as a single reagent) (L-003126-00-0005; Dharmacon).

ZONAB (YBX3): ON-TARGETplus YBX3 siRNA SMARTpool (a mixture of 4 siRNAs provided as a single reagent) (L-015793-00-0005; Dharmacon).

FOXA1: ON-TARGETplus FOXA1 siRNA SMARTpool (a mixture of 4 siRNAs provided as a single reagent) (L-010319-00-0005; Dharmacon).

### 2.3. RNA Extraction, Reverse Transcription and Quantitative Real-Time Polymerase Chain Reaction

Cells were plated in duplicate at 30,000 cells per well in 24-well plates, and gene-silenced using transient transfection as described above. Following 72 h incubation, RNA was extracted from the cells using TRI-reagent (Sigma-Aldrich, Dharmstadt, Germany) as per the manufacturer’s instructions. RNA concentration was analyzed using a Roche Nanodrop-8000, with A260/280 values > 1.8 deemed to be of sufficient quality. Genomic DNA elimination reactions were carried out, and reverse transcription was carried out using QuantiTect Reverse Transcription Kits (Qiagen, Venlo, The Netherlands) to make complementary DNA (cDNA). The cDNAs were subjected to real-time quantitative PCR (qRT-PCR) on a Roche LightCycler-480 instrument using a LightCycler-480 SYBR Green-I Master Mix (Roche, Basel, Switzerland). Primers (sequences below) targeting genomic DNA were obtained from Integrated DNA Technologies Inc (Coralville, IA, USA). RPLPO was used as a housekeeping gene, and data analysis was carried out via the delta-delta-Ct method. All treatments were performed in experimental triplicate and each sample was measured in technical duplicate.

Primer sequences:

RPLP0—Forward: GGC AGC ATC TAC AAC CCT GA

- Reverse: AAC ATT GCG GAC ACC CTC C

JAM-A—Forward: CTC TCA GTC CCC TCG CTG TA

- Reverse: AAT GCC AGG GAG CAC AAC AG

HER2—Forward: ACG TTT GAG TCC ATG CCC AA

- Reverse: AGG TAG TTG TAG GGA CAG GCA

ZONAB—Forward: GTT GAA GGA GAG AAG GGT GCA G

- Reverse: CTC CTC CTC CCC AGC GTA A

FOXA1—Forward: AGG GCT GGA TGG TTG TAT TG

- Reverse: GCT CGT AGT CAT GGT GTT CAT

### 2.4. In Silico Transcription Factor Binding Determination

Bioinformatics analysis was carried out to identify putative FOXA1 transcription factor binding sites in the proximal promoter of the HER2 gene. The datasets used were FOXA1 binding data from the ENCODE (Encyclopedia of DNA Elements) project (https://www.encodeproject.org/, accessed on 15 December 2021) and FOXA1 position-specific scoring matrices (PSSM) from TRANSFAC [26] with the TFFFIND search tool from the Piptools package [27]. The ENCODE data identified regions of the HER2 promoter which were bound by FOXA1, and TFFIND with TRANSFAC was used to localize the binding sites within these regions. Based upon this, the following oligonucleotide sequences were designed to replicate the putative FOXA1-binding promoter regions of HER2 and synthesized by Integrated DNA Technologies (with putative FOXA1 binding sequences underlined):

Oligo 1: 5′-CATGTACCCTGCTCCCTGAGTAAATAAAGCTCCTGGATGT-3′

Scramble Oligo 1:

5′-GAGTAACGCTCTGTCACCGTCAGTCGCATTAGCCTATATA-3′

Oligo 2: 5′-GTTGCAGCCCCAGCCTGTTGACTTAGAGGTCACCCTCGGA-3′

Scramble Oligo 2:

5′-GTGGTCGCCTCAGTACTCACGCAACCGTCTGGCTCGTAGA-3′

### 2.5. Experimental Protein–DNA Binding Assays

As per manufacturer instructions, EpiQuik™ Nuclear Extraction Kits (Epigentek, Farmingdale, NY, USA) were used to prepare nuclear versus non-nuclear extracts from cells that had reached 80% confluence or had been gene-silenced for 72 h. To detect the binding of FOXA1 to the above oligonucleotide sequences representing the HER2 promoter in vitro, the EpiQuik™ General Protein-DNA Binding Assay Kit (Colorimetric) (Epigentek) was used according to the manufacturer’s instructions. A FOXA1/HNF3α (D7P9B) antibody (Cell Signaling Technologies, Danvers, MA, USA) was used to detect specific protein–DNA binding. Binding Activity was calculated as delta optical density (sample—blank) × sample dilution.

### 2.6. Protein Extraction and Western Immunoblotting

Total protein extract was prepared from cells in ice-cold lysis buffer (0.1 M KCl, 2.5 mM NaCl, 3.5 mM MgCl_2_, 10 mM HEPES, 1% Triton-X100 and 1× protease/phosphatase inhibitor cocktails). Protein content was quantified via bicinchoninic assay (ThermoFisher, Waltham, MA, USA), and equivalent concentrations were separated by SDS-polyacrylamide gel electrophoresis using a Bio-Rad Mini-Protean III gel system (Bio-Rad Laboratories, Hertfordshire, UK). Resolved proteins were transferred to nitrocellulose or methanol-activated PVDF membranes at 100 V for 75 min using a Bio-Rad Mini-Protean III blotting system and Western blotted using the following antibodies: human HER2 (rabbit 58613, Cell Signaling), JAM-A (mouse anti-JAM-1, 612120, BD Biosciences, San Jose, CA, USA), FoxA1/HNF3α (D7P9B) (58613, Cell Signaling Technologies), ZONAB (rabbit 40-2800 ThermoFisher), GAPDH (mouse 0411 sc-47724 Santa Cruz Biotechnology, Dallas, TX, USA), Lamin A/C (4C11, 4777, Cell Signaling), AKT (pan, 40D4, 2920, Cell Signaling), Phospho-AKT (Ser473, 9271, Cell Signaling), Erk1/2 (p44/42 MAPK, L34F12, Cell Signaling), Phospho-Erk 1/2 (p44/42 MAPK, Thr202/Tyr204, 4370, Cell Signaling) or beta-Actin (ab8227, Abcam, Cambridge, UK). The following horseradish peroxidase-conjugated secondary antibodies were used: goat anti-rabbit IgG (7074, Cell Signaling Technologies), goat anti-mouse IgG (A9044, Sigma-Aldrich). Following incubation with Western Lightning Plus Enhanced Chemiluminescence ECL reagent, blots were imaged on a ChemiDoc XRS+ system (Bio-Rad Laboratories, Hertfordshire, UK). Membranes were stripped of the first primary antibody using a 0.7% β-mercaptoethanol solution in order to re-probe with a loading control antibody (actin) for the purposes of relative densitometry analysis (ImageJ; [28]).

### 2.7. Statistical Analysis

Averaged data from triplicate cell biology experiments are presented and were graphed along with standard error of the mean (SEM) values. Two-tailed, equal variance Student’s *t*-tests were used to determine statistical significance (* *p* < 0.05, ** *p* < 0.01, *** *p* < 0.001) between the indicated conditions for PCR and Western blot studies. For results from timer.cistrome.org [29,30] (accessed on 15 December 2021), heatmaps represent the purity-adjusted partial Spearman’s rho value as the degree of gene expressional correlation between F11R (JAM-A) and ERBB2 (HER2). For results from kmplot.com [31], patient samples were divided into low versus high expression (with the cutoff either auto-selected by the online tool or being the upper quartile, as stated in individual figure legends) of the selected genes (using expression and survival data from GEO, EGA and TCGA). Datasets used by kmplot.com for analysis are shown in Supplemental Table S1 (breast cancer) and Supplemental Table S2 (gastric cancer). Hazard ratios with 95% confidence intervals and logrank *p* values were calculated from the Kaplan–Meier curves. Throughout, values with *p* < 0.05 were considered statistically significant.

## 3. Results

### 3.1. High Gene Expression of JAM-A and HER2 Correlate in Many Cancer Types

**Figure 1:**
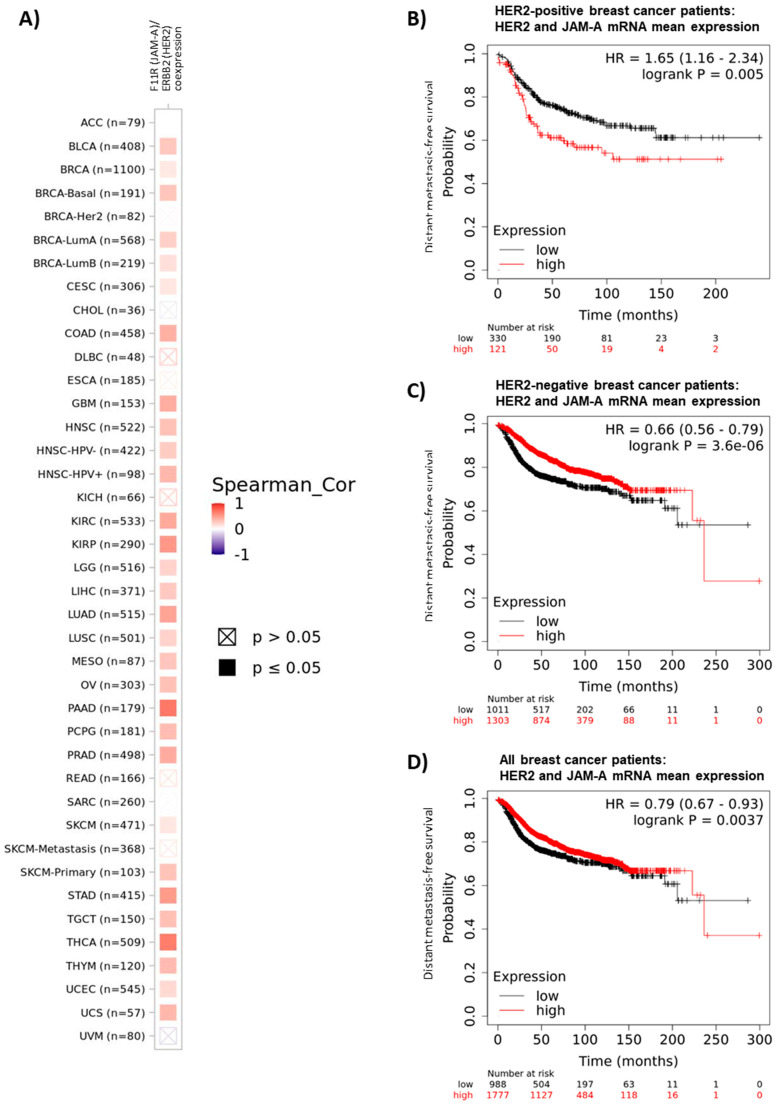
JAM-A and HER2 gene expression positively correlates in multiple cancer types and their high mean expression is associated with poor survival in HER2-positive breast cancer patients. (**A**) Co-expression of the JAM-A (F11R) and HER2 (ERBB2) genes was analyzed in multiple cancer types using the web resource TIMER 2.0: Tumor IMmune Estimation Resource (http://timer.cistrome.org/ [29,30], accessed on 3 December 2021), which incorporates > 10,000 patient samples across 40 cancer types from The Cancer Genome Atlas (TCGA) database. The heatmap represents the purity-adjusted partial Spearman’s rho value as the degree of gene expressional correlation between F11R and ERBB2 in various cancer types. (**B**–**D**) Mean mRNA expression of the JAM-A (F11R) and HER2 (ERBB2) genes in breast cancer patients was correlated with distant metastasis-free survival (DMFS) in breast cancer patient samples using the online resource kmplot.com [31], (accessed on 2 December 2021) using only JetSet probes. The cutoff between high versus low expression was auto-selected by the online tool.

High JAM-A expression is a feature of certain aggressive breast cancers, particularly HER2-positive breast cancers [3,7,8]. There are also significant positive correlations between JAM-A and HER2 gene expression in multiple other cancers, specifically 30/40 or 75% of The Cancer Genome Atlas (TCGA) subclassifications that can be interrogated on the online platform timer.cistrome.org [29,30] (accessed on 15 December 2021). These cancers are shown in Figure 1A: specifically bladder urothelial carcinoma (BLCA), breast invasive carcinoma (BRCA), basal-like breast cancer (BRCA-basal), Luminal A breast cancer (BRCA-LumA), Luminal B breast cancer (BRCA-LumB), cervical squamous cell carcinoma/endocervical adenocarcinoma (CESC), colon adenocarcinoma (COAD), brain glioblastoma multiforme (GBM), head and neck squamous cell carcinoma (HNSC), human papilloma virus-negative HNSC, human papilloma virus-positive HNSC, kidney renal clear cell carcinoma (KIRC), kidney renal papillary cell carcinoma (KIRP), brain lower grade glioma (LGG), liver hepatocellular carcinoma (LIHC), lung adenocarcinoma (LUAD), lung squamous cell carcinoma (LUSC), mesothelioma (MESO), ovarian serous cystadenocarcinoma (OV), pancreatic adenocarcinoma (PAAD), pheochromocytoma and paraganglioma (PCPG), prostate adenocarcinoma (PRAD), skin cutaneous melanoma (SKCM), skin cutaneous melanoma (SKCM-primary), stomach adenocarcinoma (STAD), testicular germ cell tumors (TGCT), thyroid carcinoma (THCA), thymoma (THYM), uterine corpus endometrial carcinoma (UCEC) and uterine carcinosarcoma (UCS) (Figure 1A). Moreover, interrogation of the online patient survival database kmplot.com [31] revealed links between high mean expression of JAM-A (F11R) and HER2 (ERBB2) mRNA and poorer distant metastasis-free survival (DMFS) in HER2-positive breast cancer patients (defined by the software plotting tool as all patients who were HER2-positive by array (Figure 1B), *p* = 0.005, upper quartile survival of 25.6 months versus 56.4 months in the high versus low expression groups, respectively), with the reverse trend in the much larger numbers of either HER2-negative patients (Figure 1C, *p* = 0.0000036, upper quartile survival of 118.82 months versus 60 months in the high versus low expression groups, respectively) or the breast cancer patient population as a whole (Figure 1D, *p* = 0.0037, upper quartile survival of 94.13 months versus 60.3 months in the high versus low expression groups, respectively). Incidentally, there were also positive correlations between high mean expression of JAM-A and HER2 and either recurrence-free survival (RFS) or overall survival (OS) in HER2-positive breast cancer patients; however, due to relatively small sample numbers with available genomic data on kmplot.com (882 patients for RFS, 420 patients for OS), these observations did not reach statistical significance (*p* = 0.05 for RFS and *p* = 0.12 for OS; data not shown).

### 3.2. HER2 Gene and Protein Expression Levels Are Sensitive to Alterations in JAM-A or FOXA1 in Breast Cancer Cells

**Figure 2:**
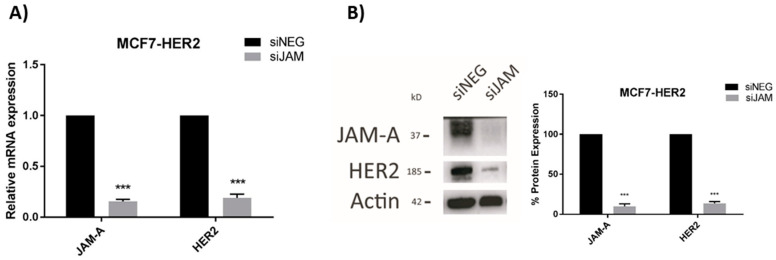

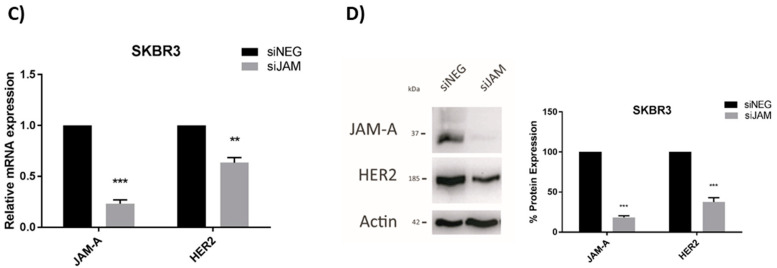
JAM-A gene silencing reduces HER2 mRNA and protein expression in HER2-positive breast cancer cell lines. SK-BR-3 and MCF7-HER2 cells were transfected with control siRNA (siNEG) or a pool of JAM-A siRNA (siJAM1 and siJAM2 combined) and harvested 72 h later for qRT-PCR and immunoblot analysis. Results show qRT-PCR analysis of JAM-A and HER2 mRNA expression after JAM-A silencing in (**A**) MCF7-HER2 and (**C**) SKBR3 cells. Representative Western blot images and densitometric analysis for HER2 and JAM-A protein normalized to actin expression after JAM-A knockdown in (**B**) MCF7-HER2 and (**D**) SKBR3 cells. Experiments were performed three times and data represent mean ± s.e.m, compared using two-tailed, equal variance Student’s *t*-tests, ** *p* < 0.01, *** *p* < 0.001.

We previously reported that JAM-A can post-translationally regulate the expression of HER2 [3] and transcriptionally regulate expression of the HER2 family member HER3 [18] in breast cancer cells. Since the latter mechanism involved expressional regulation of the transcription factor FOXA1 [18], and FOXA1 is also a putative transcription factor for HER2 [21], this study set out to establish if JAM-A also regulated HER2 expression at a transcriptional level. We first selected three representative breast cancer cell lines with either low or high expression of HER2, in which JAM-A is also well-expressed (Supplemental Figure S1). Transient JAM-A gene silencing using an siRNA pool was sufficient to significantly reduce the mRNA and protein expression of HER2 in MCF7-HER2 cells (Figure 2A,B, respectively) and SK-BR-3 cells (Figure 2C,D, respectively). This was reproducible with the two individual siRNA constructs (Supplemental Figure S2). It would have been desirable to perform rescue experiments to test if JAM-A overexpression in JAM-A-silenced cells would restore normal levels of HER2, but this was not practically possible in an experimental setting where JAM-A was only transiently silenced. However, stable overexpression of JAM-A did upregulate HER2 protein expression in two cell lines considered to be HER2-negative (MDA-MB-231 and MCF7; Supplemental Figure S3). The blots shown reflect very long exposure times to reveal HER2 expression at all, as in a direct comparison with HER2-positive cell lines, we have previously observed that both MDA-MB-231 and MCF7 cells have low to undetectable HER2 levels [3]. It is also noteworthy that stable overexpression of JAM-A in the same HER2-negative cell lines was sufficient to increase the protein levels of survival effectors such as pAKT and pERK (Supplemental Figure S4A) and to enhance cell viability (Supplemental Figure S4B). This was reproduced in HER2-negative cells using individual JAM-A siRNA constructs (Supplemental Figure S5). Whether these effects resulted from direct signaling through FOXA1 was not experimentally tested either here or in the HER2-positive cell lines; therefore, it cannot be excluded that they reflect FOXA1-independent signaling pathways downstream of JAM-A.

**Figure 3:**
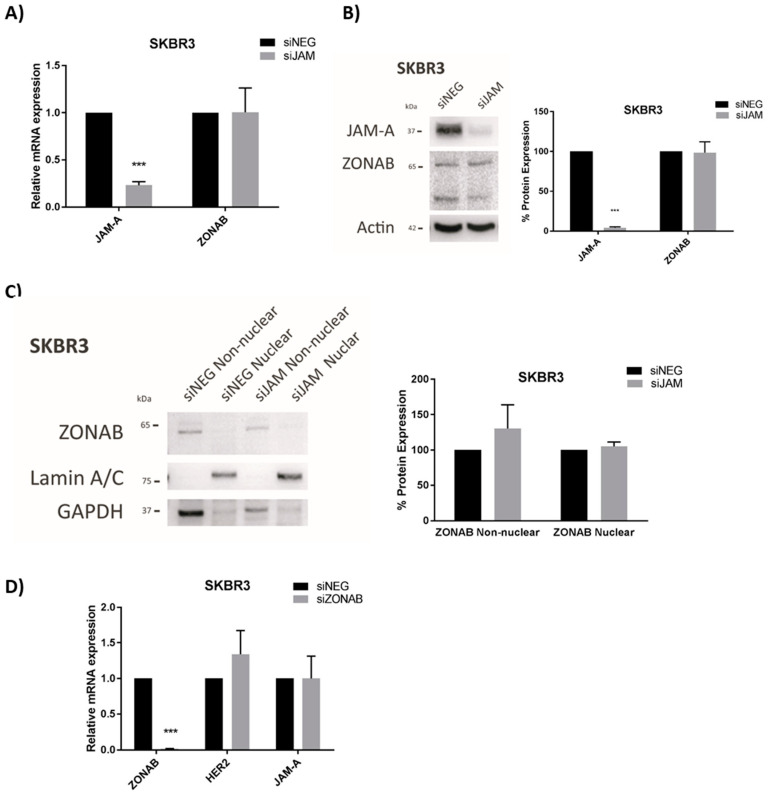
JAM-A does not regulate the HER2 transcriptional repressor YBX3/ZONAB in SK-BR-3 cells. (**A**,**B**) SKBR3 cells were transfected with control siRNA (siNEG) or a pool of JAM-A siRNA and harvested 72 h later for qRT-PCR (**A**) and immunoblot/densitometric (**B**) analysis of three independent experiments. Data represent mean ± s.e.m, compared using two-tailed, equal variance Student’s *t*-tests, *** *p* < 0.001. (**C**) SK-BR-3 breast cancer cells were transfected with 25 nM of control siRNA or a pool of JAM-A siRNA. Cells were harvested 72 h later and non-nuclear/nuclear fractions were prepared for Western blot analysis. The two bands for ZONAB reflect parallel detection of its long form and an alternately spliced shorter form. (**D**) SKBR3 cells were transfected with control siRNA or ZONAB Smartpool siRNA. After 72 h, RNA was extracted for RT-qPCR analysis. Experiments were performed three times and data represent mean ± s.e.m, compared using two-tailed, equal variance Student’s *t*-tests, *** *p* < 0.001.

The HER2 transcriptional repressor YBX3/ZONAB [32] shares a binding partner with JAM-A, namely the tight junction protein ZO-1 [33]. Therefore, we first considered the possibility that high JAM-A levels could promote ZONAB sequestration at the tight junction, restraining it from entering the nucleus where it would otherwise repress HER2 gene transcription. However, alterations in JAM-A expression levels (by gene silencing, confirmed at mRNA and protein level in Figure 3A,B, respectively) had no effect on the nuclear levels of ZONAB (Figure 3C), nor did JAM-A silencing influence either the mRNA (Figure 3A) or protein (Figure 3B) expression of ZONAB in SK-BR-3 cells. The lack of any notable nuclear signal for ZONAB may imply the absence of necessary translocation signals in this particular experimental setting, and in fact ZONAB silencing in this cell line did not even increase HER2 mRNA expression (Figure 3D). However, we cannot exclude the possibility of a long-lived ZONAB protein maintaining repression, which was echoed by the fact that ZONAB gene silencing for periods as long as 10 days was insufficient to reduce ZONAB expression at the protein level (Supplemental Figure S7). Collectively, this suggested that other mechanisms linking JAM-A to transcriptional control of the HER2 gene in our experimental setting may be more significant.

**Figure 4:**
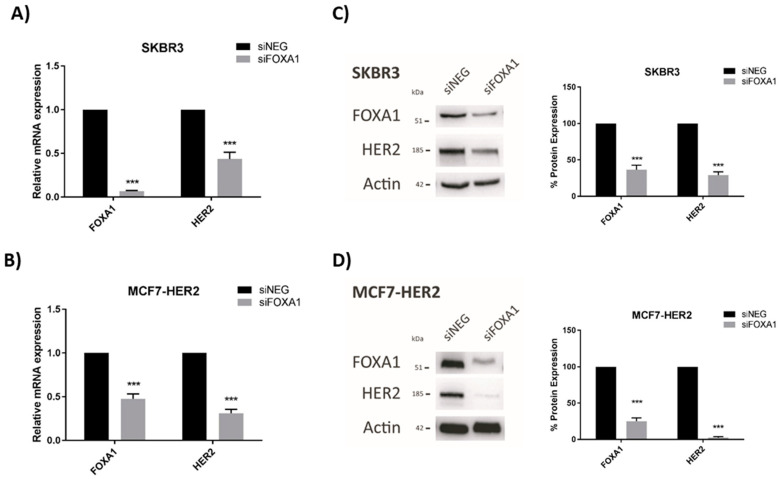
FOXA1 knockdown reduces HER2 mRNA and protein expression in breast cancer cells. SK-BR-3 and MCF7-HER2 cells were transfected with control siNEG or FOXA1 Smartpool siRNA. RNA and protein were extracted for qRT-PCR and immunoblot analysis. Results show qRT-PCR analysis of HER2 mRNA expression after FOXA1 knockdown in (**A**) SK-BR-3 and (**B**) MCF7-HER2 cells. Representative Western blot images and densitometric analysis for FOXA1 and HER2 protein normalized to actin expression after FOXA1 knockdown in (**C**) SK-BR-3 and (**D**) MCF7-HER2 cells. Experiments were performed three times and data represent mean ± s.e.m, compared using equal variance, unpaired Student’s *t*-tests, *** *p* < 0.001.

Having already established that JAM-A levels positively regulate those of the HER3 transcription factor FOXA1 in breast cancer cells [18], we tested whether FOXA1 levels directly altered HER2 expression in our cell lines. Transient gene silencing of FOXA1 was confirmed to significantly reduce HER2 mRNA expression in SK-BR-3 (Figure 4A) and MCF7-HER2 (Figure 4B) cells. FOXA1 silencing similarly exerted significant reductions in the protein expression of HER2 in SK-BR-3 (Figure 4C) and MCF7-HER2 (Figure 4D) cells. As similarly noted for Figure 2, it would have been desirable to perform rescue experiments to test if FOXA1 overexpression in FOXA1-silenced cells would restore normal levels of HER2. However, this was not possible when FOXA1 was only transiently silenced, since the time required for successful overexpression would have greatly exceeded the period of optimal silencing.

### 3.3. Characterizing FOXA1 Binding Sites in the HER2 Gene Proximal Promoter

**Figure 5:**
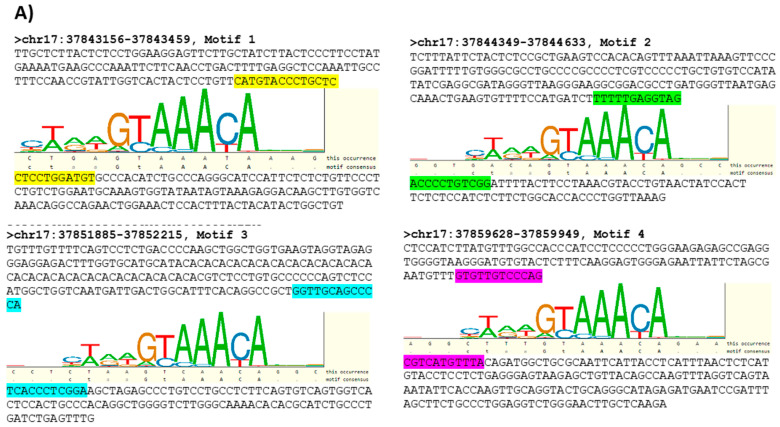

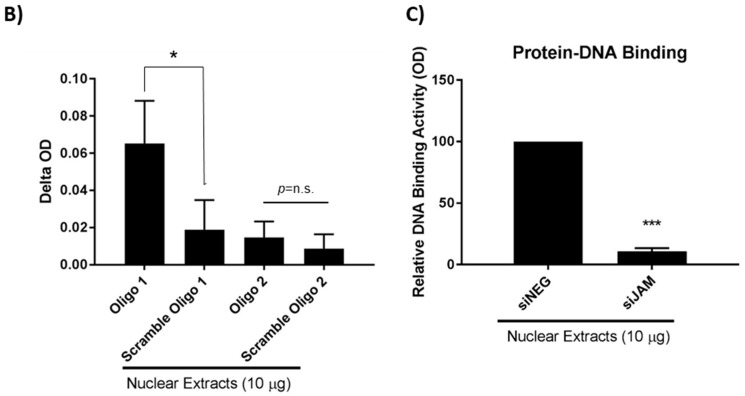
FOXA1 binds to a specific sequence within the HER2 gene promoter and its binding activity is influenced by JAM-A expression. (**A**) The ENCODE project (https://www.encodeproject.org/, accessed on 15 December 2021), TRANSFAC and the TFFFIND search tool from the Piptools package were used to identify segments of the HER2 proximal gene promoter in breast cancer cells that contained binding sequences for FOXA1. Four such sequences were identified (sequence logo for the FOXA1 consensus motif shown, accessed on 15 December 2021), of which two were synthesized as oligonucleotides for binding assays. (**B**) Nuclear proteins were extracted from untreated MCF7-HER2 cells and those transfected with non-targeting control siRNA or JAM-A siRNA, and used in protein–DNA binding assays. Nuclear protein binding activity to two different oligonucleotide sequences representing the HER2 gene promoter. (**C**) Relative optical densities of FOXA1 binding to the HER2 gene promoter in JAM-A knockdown compared to control siNEG conditions. Experiments were performed three times and data represent mean ± s.e.m, compared using equal variance, unpaired Student’s *t*-tests, * *p* < 0.05, *** *p* < 0.001.

A targeted bioinformatics analysis was next undertaken to identify putative sequences where FOXA1 binds to the HER2 gene promoter. Using data from the ENCODE (Encyclopedia of DNA Elements) project (https://www.encodeproject.org/, accessed on 15 December 2021), broad regions where FOXA1 binds to the HER2 promoter were identified by analysis of CHIP-seq information from T-47D breast cancer cells. As those regions are extensive and of impractical use for in vitro binding assays, the search for FOXA1 binding sites in the HER2 promoter was further narrowed using transcription factor binding sites (TFBS) information from TRANSFAC [26] and the PipTools package [27]. The analysis resulted in a list of four putative sequences within regions where FOXA1 is believed to bind to the HER2 gene promoter (Figure 5A; highlighted sequences). Supplemental Figure S8 shows ChIP-seq data from the ENCODE project detailing the locations of the FOXA1 binding sites in the HER2 gene promoter.

Two of these sequences were prepared as oligonucleotides to be used in protein–DNA binding assays in MCF7-HER2 cells, based on the prediction that they contained TFBS for FOXA1 and formed part of the FOXA1 target regions from the ENCODE project. As shown in Figure 5B, FOXA1 bound strongly to the region of the HER2 gene promoter represented by Oligo 1 relative to its scrambled control. In contrast, FOXA1 bound negligibly to the region of the HER2 gene promoter represented by Oligo 2. The fact that the second putative FOXA1 TFBS represented by Oligo 2 was not confirmed experimentally (Figure 5B) is not unexpected, as these are only predicted transcription factor binding sites (TFBS), based on PSSMs from TRANSFAC. The PSSMs in TRANSFAC are generated from experimentally-determined TFBS; however, they are short (the TF only touches the DNA in a narrow location) and degenerate (there is a great deal of variability allowed in the binding sequence). The TFBS predictions are therefore prone to high false-positive rates. In addition, the ENCODE project used a different breast cell line (T-47D). More importantly, JAM-A gene silencing significantly reduced FOXA1 binding to Oligo 1 compared with control (non-targeting siRNA) conditions (Figure 5C).

**Figure 6:**
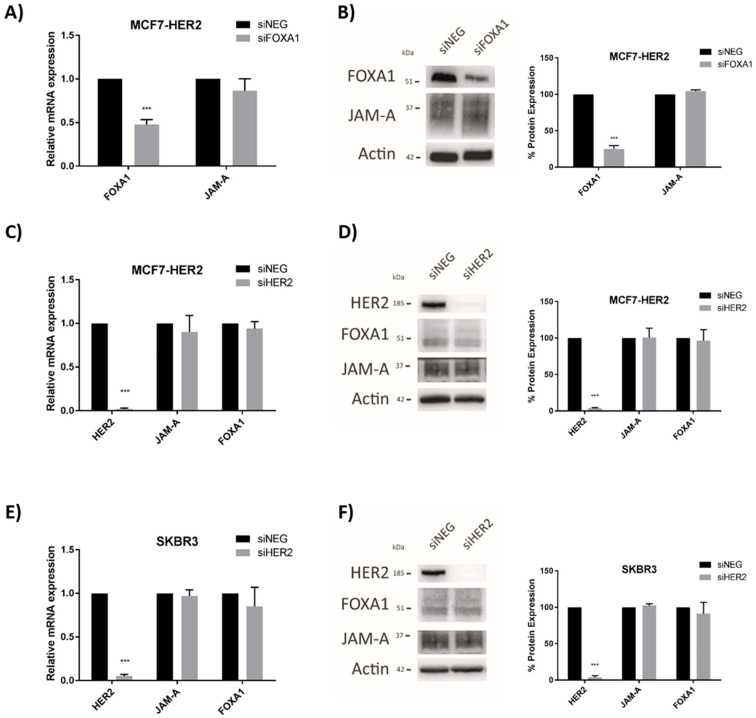
FOXA1 or HER2 gene silencing do not affect JAM-A expression in breast cancer cells. MCF7-HER2 cells were transfected with 25 nM of control siNEG, FOXA1 or HER2 Smartpool siRNA, and RNA and protein extracted for qRT-PCR and immunoblot analysis. Representative Western blot images and densitometric analysis are shown for expression of individual proteins normalized to actin expression after FOXA1 or HER2 gene silencing. FOXA1 and JAM-A mRNA (**A**) and protein (**B**) expression in MCF7-HER2 cells after FOXA1 gene silencing. mRNA (**C**) and protein (**D**) expression of FOXA1 or JAM-A in MCF7-HER2 cells after HER2 gene silencing. mRNA (**E**) and protein (**F**) expression of FOXA1 or JAM-A in SK-BR-3 cells after gene silencing of HER2. All experiments were performed three times, and data represent mean ± s.e.m, compared using equal variance, unpaired Student’s *t*-tests, *** *p* < 0.001.

This supports the view that FOXA1 downregulation secondary to JAM-A silencing would reduce the pool of FOXA1 protein available to bind the HER2 gene promoter and influence its transcription. That this pathway is unidirectional is supported by evidence that gene silencing of FOXA1 had no effect on the mRNA or protein expression of JAM-A in MCF7-HER2 cells (Figure 6A,B, respectively) or SK-BR-3 breast cancer cells (shown in the supplementary data of our recently published study [18]). Similarly, HER2 gene silencing had no effect on the mRNA (Figure 6C,E) or protein (Figure 6D,F) expression of JAM-A or FOXA1 in, respectively, MCF7-HER2 or SK-BR-3 breast cell lines. This supports a model whereby JAM-A-dependent regulation of FOXA1 and, subsequently, HER2 expression is unidirectional rather than bidirectional.

### 3.4. Coincident High Gene Expression of JAM-A, HER2 and FOXA1 Is Associated with Poor Survival in HER2-Positive Breast and Gastric Cancer Patients

**Figure 7:**
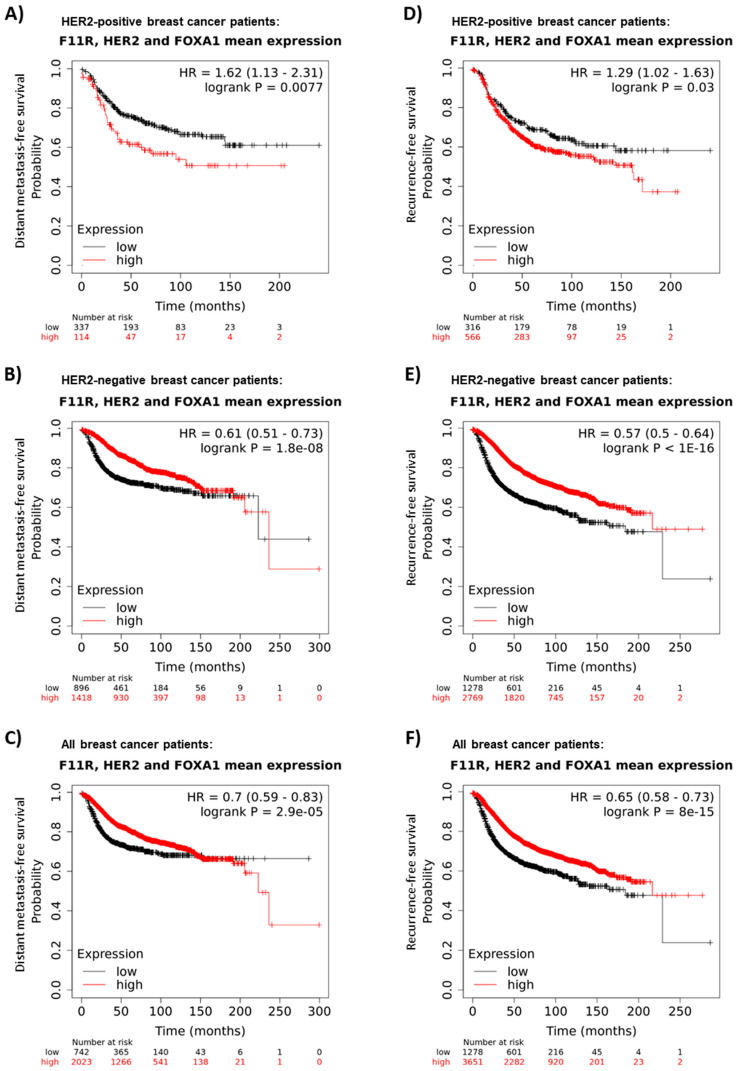
High average gene expression of JAM-A, HER2 and FOXA1 correlates with poorer survival prospects in HER2-positive but not HER2-negative breast cancer patients. Mean mRNA expression of the JAM-A (F11R), HER2 (ERBB2) and FOXA1 genes in breast cancer patients was correlated with distant metastasis-free survival (DMFS; **A**–**C**) or recurrence-free survival (RFS; **D**–**F**) in breast cancer patient samples using the online resource kmplot.com [31], (accessed on 8 December 2021) using only JetSet probes. The cutoff between high and low expression was automatically selected by the online tool.

Finally, the online database kmplot.com [31] was interrogated to examine the significance of high combined JAM-A, HER2 and FOXA1 gene expression for breast cancer patient survival. As shown in Figure 7, high mean expression of JAM-A, HER2 and FOXA1 mRNA in HER2-positive breast cancer patients (where the best cutoff between high and low expression was auto-selected by the online tool) was associated with significantly poorer distant metastasis-free survival (DMFS; Figure 7A, upper quartile survival of 25.8 months versus 53.45 months for patients with low combined expression) and recurrence-free survival (RFS; Figure 7D, upper quartile survival of 29.33 months versus 38.57 months for patients with low combined expression). These trends were specific to the HER2-positive patient population, since HER2-negative patients had a negative correlation between high co-expression of JAM-A, HER2 and FOXA1 and DMFS (Figure 7B; median survival of 236.22 months versus 222.81 months for patients with low combined expression) and a similar negative correlation for RFS (Figure 7E; median survival of 216.66 months versus 184.04 months for patients with low combined expression). In support of these findings, data from all patients combined (both HER2-positive and HER2-negative) revealed a similar negative correlation between high co-expression of JAM-A, HER2 and FOXA1 and DMFS (Figure 7C; upper quartile survival of 95.64 months versus 38.5 months for patients with low combined expression). There was also a similar negative correlation for RFS in the whole patient population (Figure 7F; median survival of 216.66 months for patients with high mean expression of JAM-A, HER2 and FOXA1 versus 184.04 months for patients with low combined expression). Incidentally, the addition of FOXA1 to our analysis (over and above the combination of mean JAM-A and HER2 mRNA expression) had an interesting impact on overall survival (OS) figures in breast cancer patients, with high mean expression of JAM-A, HER2 and FOXA1 achieving a statistically significant positive correlation with poorer overall survival in both HER2-positive patients and the entire breast cancer patient population, alongside a significant negative correlation in HER2-negative patients (Supplemental Figure S9). Specifically, upper quartile OS for the high versus low expression cohorts was 43 vs. 63 months (HER2-positive patients), 124 vs. 70 months (HER2-negative patients) or 66 vs. 115 months (all patients). The significance of these findings was not confined to breast cancer patients, since overall survival (OS) in HER2-positive gastric cancer patients was also significantly lower if patients expressed high combined mean mRNA levels of JAM-A, HER2 and FOXA1 (Supplemental Figure S6A). There was an opposite OS trend in HER2-negative patients (Supplemental Figure S6B), while all gastric cancer patients combined showed a significant positive correlation between high mean JAM-A, HER2 and FOXA1 expression and poor OS (Supplemental Figure S6C) similar to that in the HER2-positive population alone. Collectively, these data highlight important functional impacts of a possible JAM-A, HER2 and FOXA1 axis on cancer patient outcomes.

## 4. Discussion

Junctional Adhesion Molecule-A (JAM-A) is a transmembrane adhesion protein of the immunoglobulin superfamily of proteins, and the founding member of the JAM family of glycoproteins [34]. Published reports on the contribution of JAM-A to cancer development, although sometimes controversial, have been well-summarized in a recent review [35]. As the principal role of JAM-A is in cellular adhesion, one might predict that loss of JAM-A would promote local invasion, increasing the risk of distant metastasis and correlating with poor patient prognosis in general. In the context of breast cancer, one early study made this connection [36]. However, more recent studies with larger patient populations have convincingly shown the opposite, namely that high expression of JAM-A predicts early tumor recurrence and reduced life expectancy in breast cancer patients [3,7,8]. In fact, a review from our laboratory has highlighted intriguing evidence implicating gain rather than loss of multiple adhesion proteins in cancer initiation and progression [37].

The current investigations stemmed from observations that JAM-A and HER2 are co-expressed in breast cancer cells and patient tissues [3], that cleavage of overexpressed JAM-A may be a biomarker of resistance to HER2-targeted therapies in breast cancer patients [13], and that JAM-A acts as a novel transcription-level regulator of the HER2 family member HER3 in breast cancer settings [18]. Previous findings that high JAM-A and HER2 are associated with poor prognosis in breast cancer patients [3,7,8] were supported in two different ways: (1) by revealing via an online survival tool [38] that high co-expression of JAM-A and HER2 mRNA associate with poor survival outcomes in breast cancer patients, and (2) by providing data that expressional changes in JAM-A induce parallel changes in HER2 expression in breast cancer cell lines.

Although there was prior evidence that JAM-A could regulate HER2 expression at post-translational levels via either the proteasome [3] or lysosomes [15], our findings in the current study provided the first proof that JAM-A could also regulate HER2 at a transcriptional level. We postulated, based on likely mechanisms of gene regulation [39], that this involved JAM-A interference with a transcriptional factor, which either promoted or repressed HER2 gene expression [40]. A transcriptional repressor of the HER2 gene, ZONAB, was first considered since it physically associates with a JAM-A binding partner called ZO-1 [41,42]. Previous studies have shown that abundant cellular levels of ZO-1 sequester ZONAB at the tight junction complex, preventing it from translocating into the nucleus and repressing transcription of the HER2 gene [32,43]. However, the current study could not confirm an influence of JAM-A upon ZO-1 or ZONAB expression or localization in the cell lines tested, and attempts to link ZONAB association to HER2 gene repression remained fruitless.

Turning next to the possibility that JAM-A regulates HER2 gene expression, the transcription factor FOXA1 was considered since it has recently been described as a downstream effector of JAM-A in transcriptionally regulating the HER2 family member HER3 [19,20]. FOXA1 is the founding member of the forkhead box (FOX) family of transcription factors. FOXA1/HNF3α, FOXA2/HNF3β and FOXA3/HNF3γ constitute the FOXA subfamily, which were originally named hepatocyte nuclear factors (HNFs) due to their regulation of liver-specific gene expression [44,45]. FOXAs have been found to regulate many genes involved in developmental specification of not just hepatic but also several other tissues [46].

In breast cancer, overexpression of FOXA1 and HER2 has already been demonstrated to be strongly associated with estrogen receptor (ER)-negative breast tumors [21]. Our gene expression/survival correlation data revealed that high co-expression of HER2, JAM-A and FOXA1 mRNA was associated with poorer survival characteristics (both distant metastasis-free survival and recurrence-free survival) in HER2-positive breast cancer patients. The importance of this connection between JAM-A expression and HER2-positivity is reinforced by the fact that HER2-negative patients and the overall breast cancer patient population showed the opposite correlation, namely more favorable survival statistics in those with high coincident JAM-A, HER2 and FOXA1 expression. It is also noteworthy that while high JAM-A and HER2 co-expression was only on the boundary of a statistically significant correlation with poor recurrence-free survival or overall survival in HER2-positive breast cancer patients, addition of FOXA1 into the analysis was sufficient to tip this into statistical significance. Of further interest is the fact that the correlation between high mean expression of JAM-A, HER2 and FOXA1 and poor patient survival in specifically HER2-positive patients is not limited to breast cancer, but also holds true for HER2-positive gastric cancer patients. Since HER2-targeted therapies have been approved for use in patients with advanced HER2-positive gastric cancer [47], this illustrates the potential clinical importance of using JAM-A as an alternative route to target HER2 expression in multiple tumor settings. Incidentally, it would have been interesting to explore the relationship of high versus low JAM-A/HER2/FOXA1 expression with various clinicopathological factors, but this information was not available in the online tools used for analysis.

Regarding potential mechanisms whereby FOXA1 regulates HER2 expression, FOXA1 has been shown to regulate HER2 expression by either CREB1 and c-Fos regulation of FOXA1 transcription or AP2α-dependent regulation of both FOXA1 and HER2 expression [21] in molecular apocrine breast cancer cells (which are estrogen receptor- and progesterone receptor-negative but have high expression of androgen receptors and FOXA1) [48,49]. It is also possible that FOXA binds to nucleosomes and induces an open chromatin configuration [50], enabling transcriptional activation either by direct recruitment of transcription initiation machinery or through indirect recruitment of other transcriptional modulators such as members of the nuclear hormone receptor superfamily [51]. However, our study found direct binding of FOXA1 to a sequence derived from the HER2 gene promoter, in a manner sensitive to FOXA1 loss. However, while it has been speculated that FOXA1 likely functions independently of co-factor recruitment in initiating transcription, this has not been experimentally verified [52]. Nor did our study provide direct experimental verification that FOXA1 binds to the specified sequence of the HER2 gene promoter in live cells, and that its binding is sensitive to JAM-A loss. Although FOXA1 binding to the HER2 gene promoter has been verified by others, and our combination of bioinformatic analysis and oligonucleotide binding assays supported such findings, it would nonetheless have been valuable to perform chromatin immunoprecipitation assays alongside an assessment of chromatin activation status for incontrovertible experimental proof.

**Figure 8:**
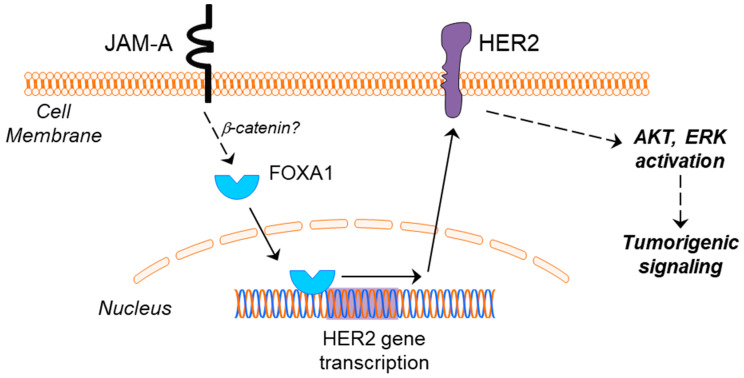
Proposed model linking JAM-A and HER2 in breast cancer. High JAM-A expression in the cell membrane of certain cancer cells leads to upregulated expression of the transcription factor FOXA1, by a pathway potentially involving β-catenin (dotted line). FOXA1 is capable of translocating to the nucleus and turning on the transcription of HER2, whereupon a subsequent upregulation of HER2 protein expression would be predicted to drive tumorigenic signaling via pathways including the activation of AKT and ERK.

Most importantly, data from this report taken together with other recent work [18] point to a unidirectional pathway whereby JAM-A levels influence FOXA1 and, subsequently, HER2 expression in breast cancer settings (Model, Figure 8). Although our other work implicated β-catenin as the link between JAM-A and the regulation of FOXA1 expression [18], we did not specifically examine whether β-catenin signaling was involved in the current pathway regulating HER2 expression downstream of JAM-A levels. However, it is reasonable to assume that, since JAM-A-dependent upregulation of FOXA1 is upstream of the latter’s entry into the nucleus, pro-transcription effects on both HER2 and HER3 could be expected. Circumstantial evidence of a connection between JAM-A, β-catenin, FOXA1 and HER2 in breast cancer patients is presented in Supplemental Table S3, where high mean expression of all four genes correlated with poorer overall survival in HER2-positive and all patients combined, but not in HER2-negative patients. Whether FOXA1 would preferentially bind to the HER2 versus HER3 genes downstream of JAM-A levels has not been investigated here, and is beyond the scope of the current study. A thorough interrogation of the potential impact of JAM-A on other factors influencing gene transcription (e.g., nuclear coactivators or corepressors) in various patient clinicopathological contexts would, however, be interesting in future studies. On a broader level, this study complements evidence that adhesion proteins should not be considered simply in terms of their mechanical adhesion properties, since (for instance) overexpression of the tight junction protein Claudin-1 in colon cancer has been shown to promote expression of ZEB1, a transcription factor which represses E-cadherin expression [53] and can, therefore, promote epithelial to mesenchymal transition. Taken together with the fact that JAM-A may also regulate HER2 stability at post-translational levels (both proteasomal [3] and lysosomal [15]), this supports the utility of JAM-A as an excellent potential cancer drug target. Indeed, an inhibitory monoclonal antibody directed against JAM-A has shown promise in reducing tumor progression in murine models of breast and skin cancer [54], a JAM-A peptide antagonist has reduced transendothelial migration of breast cancer cells [55], and an antibiotic that degrades JAM-A has displayed cytotoxicity against primary breast cells and inhibited growth in a chick embryo xenograft model of breast cancer [15].

## 5. Conclusions

In conclusion, this work has described a novel mechanism whereby JAM-A transcriptionally regulates HER2 expression through FOXA1, and revealed an important functional relevance of JAM-A, HER2 and FOXA1 co-expression for HER2-positive cancer patient survival. Future studies in this space, including extension to the several other cancers in which JAM-A is overexpressed, may prove valuable as a source of fundamental mechanistic knowledge in addition to new targeted therapies for patients.

## Data Availability

Data are contained within the article or Supplementary Material, and can be further discussed by contacting the corresponding author. The following publicly available datasets were interrogated during this study: http://timer.cistrome.org/, https://kmplot.com/analysis/index.php?p=service&cancer=breast, https://www.encodeproject.org/, https://genexplain.com/transfac/, http://genome.ucsc.edu/ (accessed on 15 December 2021).

## Supplementary Materials

The following supporting information can be downloaded at: https://www.mdpi.com/article/10.3390/cells11040735/s1, Figure S1: HER2 expression in breast cancer cell lines. 150,000 MCF7-HER2 or SKBR3 cells were plated per well in 6-well plates and whole cell lysates prepared at confluency for analysis by Western immunoblotting. Representative Western blot images and densitometric analysis of three independent experiments confirmed that MCF7-HER2 and SKBR3 cells had higher HER2 protein expression than MCF7 control cells. JAM-A was robustly expressed in all three cell lines, with the highest expression in MCF7 cells. Results were compared using two-tailed, equal variance Student’s *t*-tests, ** *p* < 0.01, *** *p* < 0.001. Figure S2: JAM-A silencing using individual siRNA constructs reduces HER2 protein expression. 150,000 MCF7-HER2 cells were plated in 6-well plates, transfected 24 h later with 25 nM control siRNA (siNEG) or JAM-A siRNA (siJAM1 or siJAM2) and harvested 72 h later for immunoblot analysis. Representative blots and densitometric analysis of three independent experiments revealed that JAM-A silencing with either siJAM1 or siJAM2 exerted similar reductions in HER2 protein expression. Data represent mean ± s.e.m, compared using two-tailed, equal variance Student’s *t*-tests, ** *p* < 0.01, *** *p* < 0.001. Figure S3: JAM-A overexpression upregulates HER2 protein expression in HER2-negative cell lines. MCF7 (A) or MDA-MB-231 (B) cells expressing either a pcDNA3 empty vector (EV) or full-length human JAM-A (WT) were grown to confluence and subjected to SDS-PAGE and Western blot analysis to compare JAM-A and HER2 protein expression. As shown in one of three representative experiments, HER2 protein expression was increased in JAM-A overexpressing cells relative to empty vector controls. Densitometric quantification of two blots per protein (relative to actin expression) is shown to the right of each image. Since HER2 was undetectable in EV-expressing MCF7 cells, it was not possible to generate a densitometric graph comparing HER2 expression against that in WT JAM-A-overexpressing cells. Figure S4: JAM-A overexpression increases cell survival in HER2-negative breast cancer cell lines. (A) 150,000 MCF7 or MDA-MB-231 breast cancer cells overexpressing JAM-A or empty vector control were plated in 6-well plates and whole cell lysates prepared at confluency for Western immunoblotting. Representative Western blot images and densitometric analysis for JAM-A protein expression normalized to actin, pAKT protein expression normalized to total AKT and pERK expression normalized to total ERK in MDA-MB-231 empty vector control and JAM-A-overexpressing cells and MCF7 empty vector control and JAM-A-overexpressing cells. Results revealed increased phosphorylation of AKT and ERK in MDA-MB-231-JAM+ and MCF7-JAM+ cells compared to controls. Experiments were performed three times and data represent mean ± s.e.m. Results were compared using two-tailed, equal variance Student’s *t*-tests, ** *p* < 0.01, *** *p* < 0.001. (B) 1500 MCF7 or MDA-MB-231 cells overexpressing JAM-A or empty vector control were seeded in 96 well-plates and cell viability measured by MTT assays at 24 h to 120 h. MDA-MB-231-JAM+ and MCF7-JAM+ cells proliferated more than empty vector control cells. Experiments were performed three times and data represent mean ± s.e.m, compared using two-tailed, equal variance Student’s *t*-tests, * *p* < 0,05, ** *p* < 0.01, *** *p* < 0.001. Figure S5: Independent JAM-A siRNA constructs reduce cell viability and survival pathway signaling in HER2-negative cells. MCF7 cells were transfected with 25 nM control siRNA (siNEG) or two different JAM-A siRNAs (siJAM1 or siJAM2) and analyzed 72 h later. (A) JAM-A gene silencing revealed that siJAM1 and siJAM2 significantly reduced cell viability as measured by Alamar blue assays. (B) Representative Western blot images and densitometry analysis for pAKT and pERK after JAM-A knockdown showed reductions in both phospho-proteins following siJAM1 or siJAM2 exposure compared to control conditions. Experiments were performed three times and data represent mean ± s.e.m, results were compared using equal variance, unpaired Student’s *t*-tests, * *p* < 0.05, ** *p* < 0.01, *** *p* < 0.001. Figure S6: High mean expression of JAM-A, HER2 and FOXA1 correlates with poorer overall survival in HER2-positive but not HER2-negative gastric cancer patients. Mean combined mRNA expression of the JAM-A (F11R), HER2 (ERBB2) and FOXA1 genes in gastric cancer patients was correlated with overall survival (OS) using the online resource kmplot.com (Szasz AM et al., Oncotarget. 2016, 7, 49322–49333; accessed on 8 December 2021), using only JetSet probes. The cutoff between high versus low expression was auto-selected by the online tool. There was a significant positive correlation between high mean expression of the three genes and poorer OS in HER2-positive gastric cancer patients ((A); median survival 20.03 versus 23.6 months for patients with high versus low expression, respectively) and the entire gastric cancer patient population ((C) median survival 22 versus 34.37 months for patients with high versus low expression, respectively). In contrast, there was a trend towards a negative correlation between high mean expression of JAM-A, HER2 and FOXA1 (combined) and poorer OS in HER2-negative patients ((B); median survival 40.7 versus 28.8 months for patients with high versus low expression, respectively). Figure S7: ZONAB silencing in SK-BR-3 cells had no effect on ZONAB protein expression. SK-BR-3 cells were plated at 150,000 cells per well in 6- well plates and transfected after 24 h with 25 nM of control siRNA or ZONAB siRNA, and repeated every 72 h. Protein was extracted at 6 days, 8 days or 10 days for Western blot analysis. Representative Western blot images and densitometric analysis of ZONAB protein expression normalized to GAPDH revealed that ZONAB silencing had no significant effect on ZONAB protein expression. Experiments were performed three times and data represent mean ± s.e.m, compared using two-tailed, equal variance Student’s *t*-tests. Figure S8: ChIP-seq binding sites for FOXA1 from the ENCODE project. A screenshot from http://genome.ucsc.edu/ (accessed on 15 December 2021) shows details of the ChIP-seq binding site for FOXA1 from the ENCODE project in the proximal promoter of HER2 (chr17:37,844,393–37,884,915) 1237 bp upstream from the transcription start site. (A) The exact location of the TFBS motif (chr17:37,843,298–37,843,314) in the 304 bp ChIP-seq region (chr17:37,843,156–37,843,459) is shown along with motif logo. (B) The exact location of the TFBS motif (chr17:37844536–37844552) in the 285 bp ChIP-seq region (chr17:37,844,349–37,844,633) is shown along with motif logo. (C) The exact location of the TFBS motif (chr17:37,852,087–37,852,103) in the 331 bp ChIP-seq region (chr17:37,851,885–37,852,215) is shown along with motif logo. (D) The exact location of the TFBS motif (chr17:37,859,747–37,859,763) in the 322 bp ChIP-seq region (chr17:37,859,628–37,859,949) is shown along with motif logo. Figure S9: High average gene expression of JAM-A, HER2 and FOXA1 correlates with poorer overall survival in HER2-positive but not HER2-negative breast cancer patients. Mean mRNA expression of the JAM-A (F11R), HER2 (ERBB2) and FOXA1 genes in breast cancer patients was correlated with overall survival (OS) in breast cancer patients using the online resource kmplot.com [31], (accessed on 8 December 2021) using only JetSet probes. The cutoff between high and low expression was automatically selected by the online tool. There was a statistically significant positive correlation between high mean expression of JAM-A, HER2 and FOXA1 and poorer OS in HER2-positive patients (A), or all patients combined (C), and a significant negative correlation between their high mean expression and poorer OS in HER2-negative patients (B). Table S1: Breast cancer datasets included in www.kmplot.com (accessed on 15 December 2021) survival analysis. Table S2: Gastric cancer datasets included in www.kmplot.com (accessed on 15 December 2021) survival analysis. Table S3: High mean mRNA expression of JAM-A, β-catenin, FOXA1 and HER2 correlates with poorer overall survival in HER2-positive but not HER2-negative breast cancer patients.
