# Supplementary material for: A Transcriptional Link between HER2, JAM-A and FOXA1 in Breast Cancer
Source: Cells. 2022 Feb 19;11(4):735. doi: 10.3390/cells11040735 (PMC8870165; doi:10.3390/cells11040735)
Supplement: Supplementary file 1 [file cells-11-00735-s001.zip › Cruz et al_SUPPLEMENTAL figs_proofs.pptx]

## Slide 1
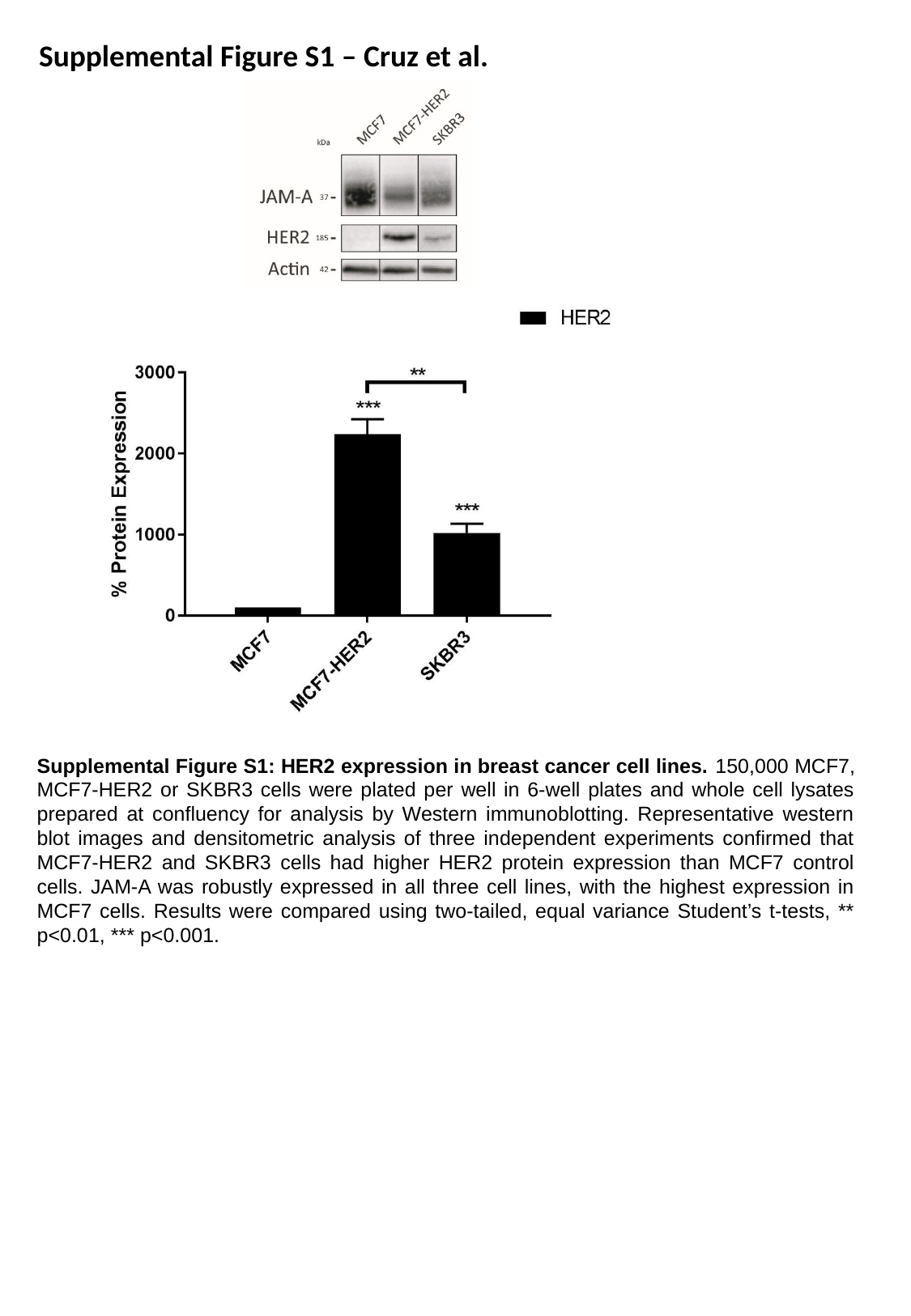

Supplemental Figure S1 – Cruz et al.
Supplemental Figure S1: HER2 expression in breast cancer cell lines. 150,000 MCF7, MCF7-HER2 or SKBR3 cells were plated per well in 6-well plates and whole cell lysates prepared at confluency for analysis by Western immunoblotting. Representative western blot images and densitometric analysis of three independent experiments confirmed that MCF7-HER2 and SKBR3 cells had higher HER2 protein expression than MCF7 control cells. JAM-A was robustly expressed in all three cell lines, with the highest expression in MCF7 cells. Results were compared using two-tailed, equal variance Student’s t-tests, ** p<0.01, *** p<0.001.

## Slide 2
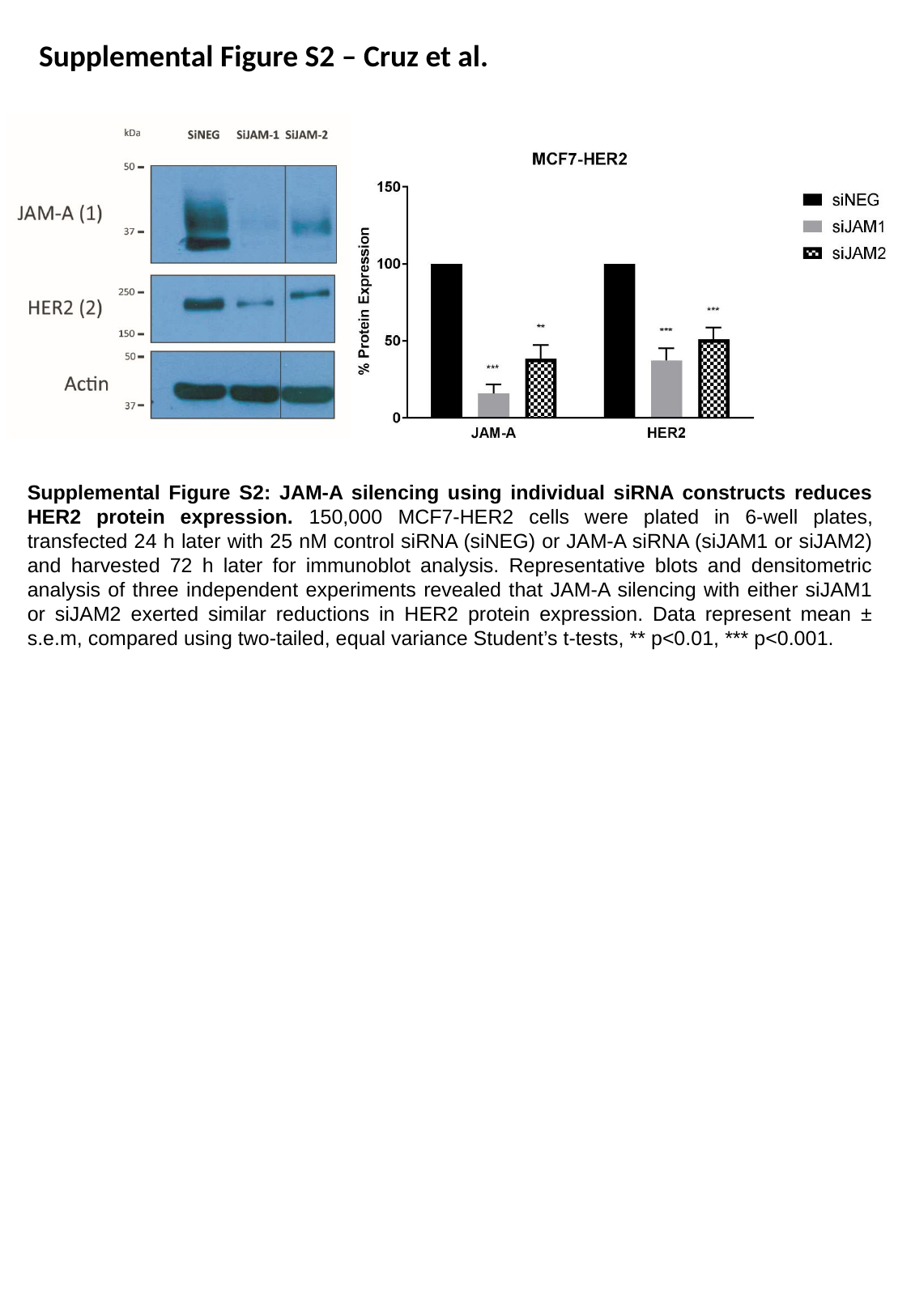

Supplemental Figure S2 – Cruz et al.
Supplemental Figure S2: JAM-A silencing using individual siRNA constructs reduces HER2 protein expression. 150,000 MCF7-HER2 cells were plated in 6-well plates, transfected 24 h later with 25 nM control siRNA (siNEG) or JAM-A siRNA (siJAM1 or siJAM2) and harvested 72 h later for immunoblot analysis. Representative blots and densitometric analysis of three independent experiments revealed that JAM-A silencing with either siJAM1 or siJAM2 exerted similar reductions in HER2 protein expression. Data represent mean ± s.e.m, compared using two-tailed, equal variance Student’s t-tests, ** p<0.01, *** p<0.001.

## Slide 3
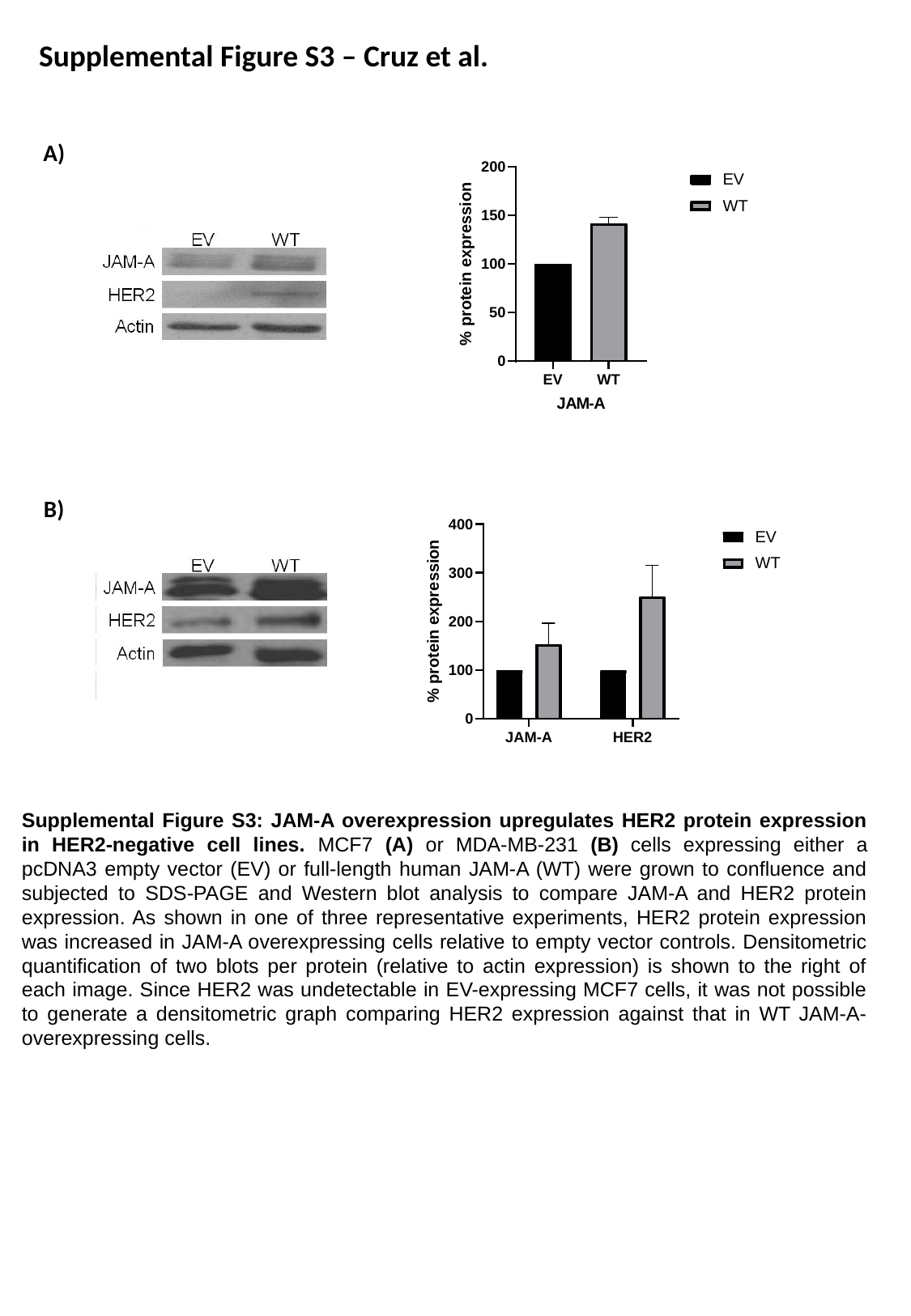

Supplemental Figure S3 – Cruz et al.
A)
B)
Supplemental Figure S3: JAM-A overexpression upregulates HER2 protein expression in HER2-negative cell lines. MCF7 (A) or MDA-MB-231 (B) cells expressing either a pcDNA3 empty vector (EV) or full-length human JAM-A (WT) were grown to confluence and subjected to SDS-PAGE and Western blot analysis to compare JAM-A and HER2 protein expression. As shown in one of three representative experiments, HER2 protein expression was increased in JAM-A overexpressing cells relative to empty vector controls. Densitometric quantification of two blots per protein (relative to actin expression) is shown to the right of each image. Since HER2 was undetectable in EV-expressing MCF7 cells, it was not possible to generate a densitometric graph comparing HER2 expression against that in WT JAM-A-overexpressing cells.

## Slide 4
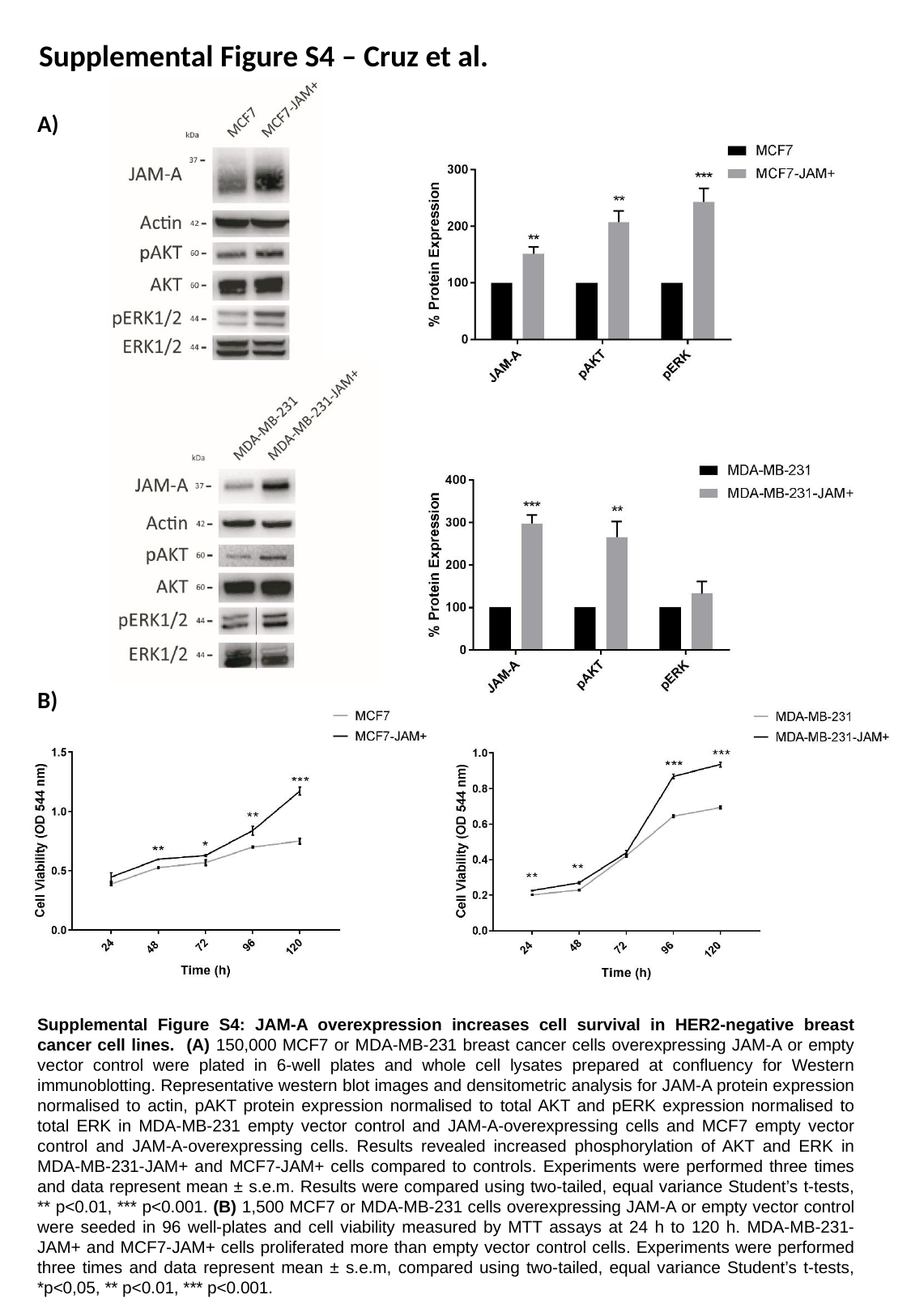

Supplemental Figure S4 – Cruz et al.
A)
B)
Supplemental Figure S4: JAM-A overexpression increases cell survival in HER2-negative breast cancer cell lines. (A) 150,000 MCF7 or MDA-MB-231 breast cancer cells overexpressing JAM-A or empty vector control were plated in 6-well plates and whole cell lysates prepared at confluency for Western immunoblotting. Representative western blot images and densitometric analysis for JAM-A protein expression normalised to actin, pAKT protein expression normalised to total AKT and pERK expression normalised to total ERK in MDA-MB-231 empty vector control and JAM-A-overexpressing cells and MCF7 empty vector control and JAM-A-overexpressing cells. Results revealed increased phosphorylation of AKT and ERK in MDA-MB-231-JAM+ and MCF7-JAM+ cells compared to controls. Experiments were performed three times and data represent mean ± s.e.m. Results were compared using two-tailed, equal variance Student’s t-tests, ** p<0.01, *** p<0.001. (B) 1,500 MCF7 or MDA-MB-231 cells overexpressing JAM-A or empty vector control were seeded in 96 well-plates and cell viability measured by MTT assays at 24 h to 120 h. MDA-MB-231-JAM+ and MCF7-JAM+ cells proliferated more than empty vector control cells. Experiments were performed three times and data represent mean ± s.e.m, compared using two-tailed, equal variance Student’s t-tests, *p<0,05, ** p<0.01, *** p<0.001.

## Slide 5
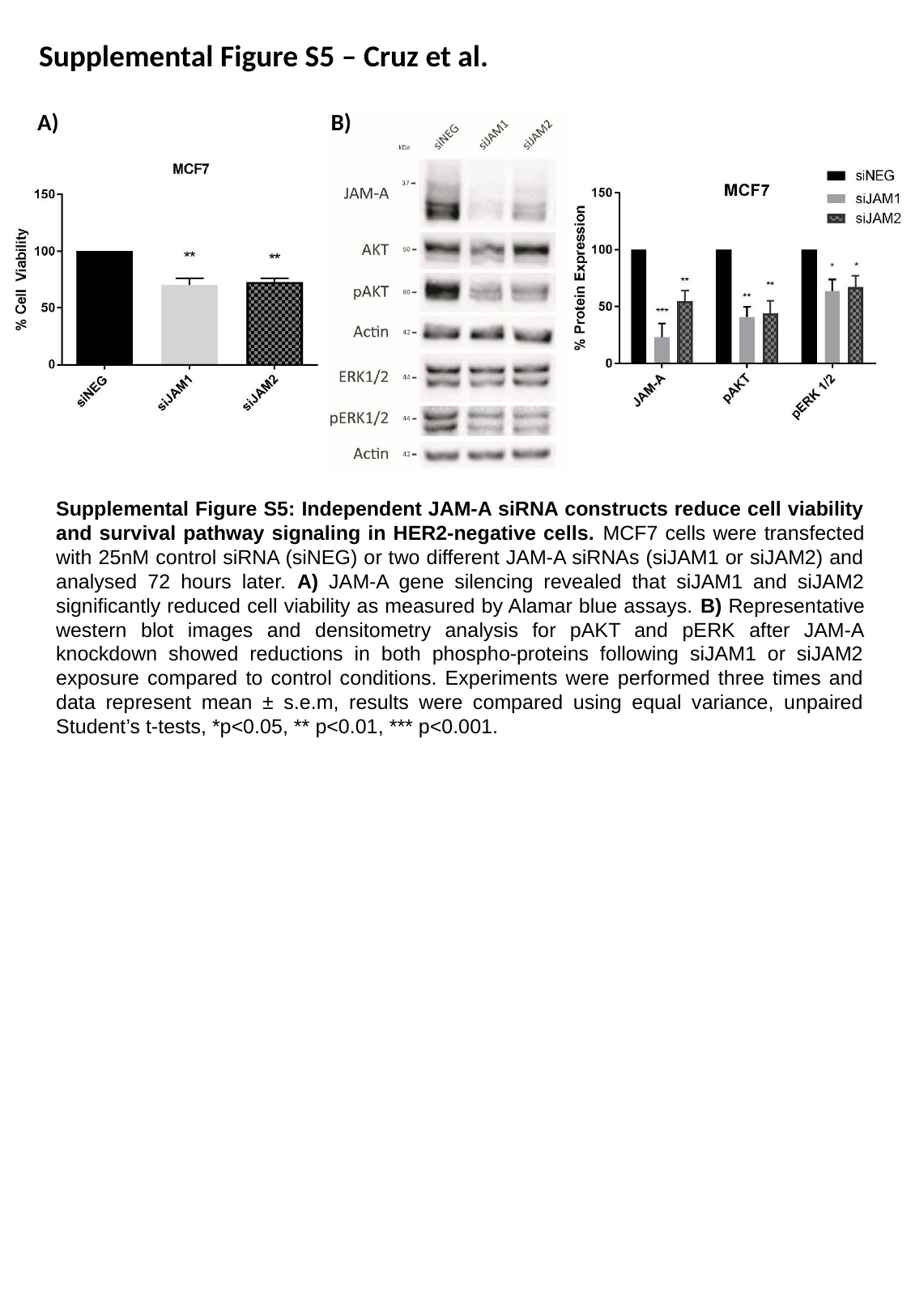

Supplemental Figure S5 – Cruz et al.
A)
B)
Supplemental Figure S5: Independent JAM-A siRNA constructs reduce cell viability and survival pathway signaling in HER2-negative cells. MCF7 cells were transfected with 25nM control siRNA (siNEG) or two different JAM-A siRNAs (siJAM1 or siJAM2) and analysed 72 hours later. A) JAM-A gene silencing revealed that siJAM1 and siJAM2 significantly reduced cell viability as measured by Alamar blue assays. B) Representative western blot images and densitometry analysis for pAKT and pERK after JAM-A knockdown showed reductions in both phospho-proteins following siJAM1 or siJAM2 exposure compared to control conditions. Experiments were performed three times and data represent mean ± s.e.m, results were compared using equal variance, unpaired Student’s t-tests, *p<0.05, ** p<0.01, *** p<0.001.

## Slide 6
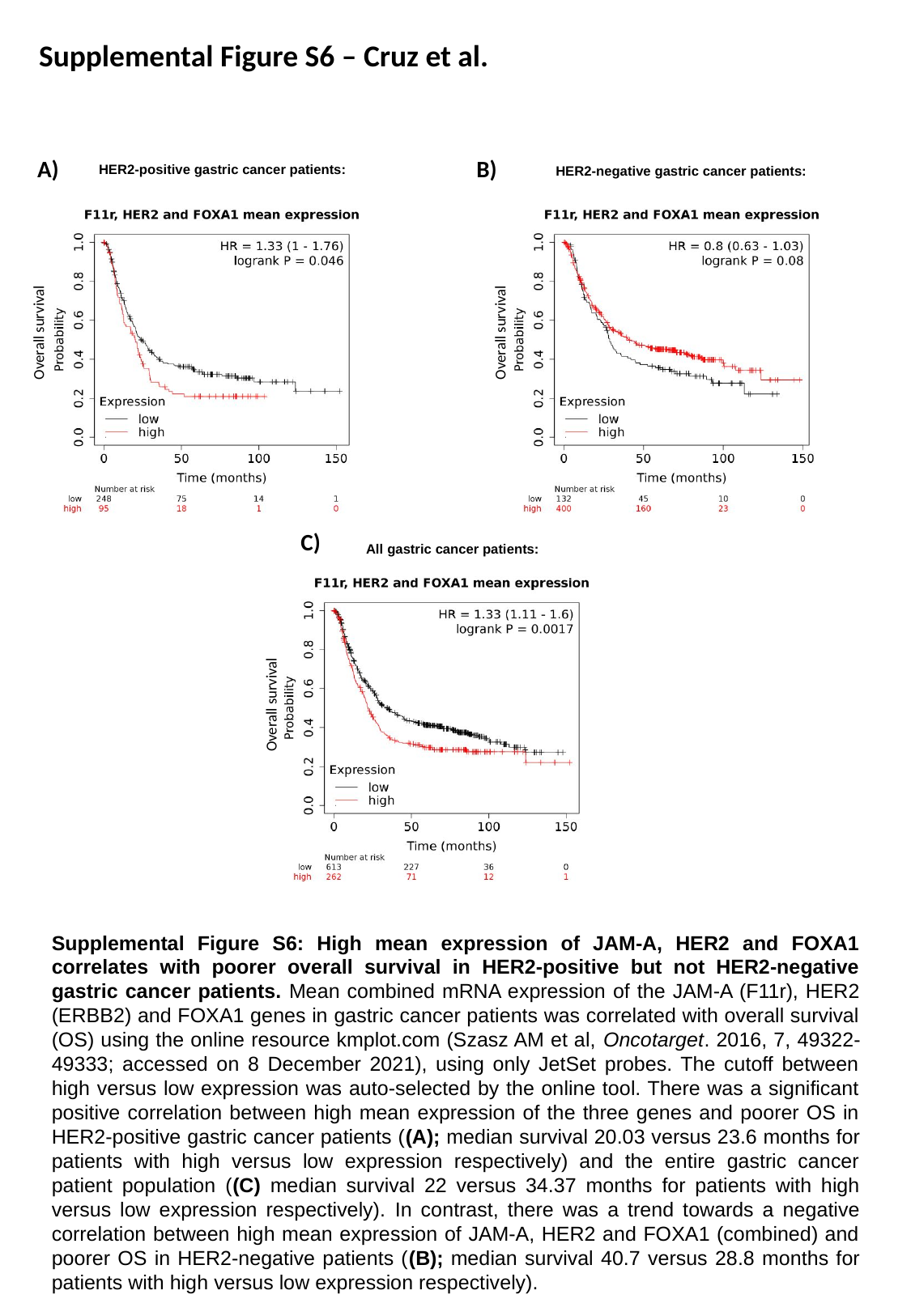

Supplemental Figure S6 – Cruz et al.
A)
HER2-positive gastric cancer patients:
B)
HER2-negative gastric cancer patients:
Overall survival
Overall survival
C)
All gastric cancer patients:
Overall survival
Supplemental Figure S6: High mean expression of JAM-A, HER2 and FOXA1 correlates with poorer overall survival in HER2-positive but not HER2-negative gastric cancer patients. Mean combined mRNA expression of the JAM-A (F11r), HER2 (ERBB2) and FOXA1 genes in gastric cancer patients was correlated with overall survival (OS) using the online resource kmplot.com (Szasz AM et al, Oncotarget. 2016, 7, 49322-49333; accessed on 8 December 2021), using only JetSet probes. The cutoff between high versus low expression was auto-selected by the online tool. There was a significant positive correlation between high mean expression of the three genes and poorer OS in HER2-positive gastric cancer patients ((A); median survival 20.03 versus 23.6 months for patients with high versus low expression respectively) and the entire gastric cancer patient population ((C) median survival 22 versus 34.37 months for patients with high versus low expression respectively). In contrast, there was a trend towards a negative correlation between high mean expression of JAM-A, HER2 and FOXA1 (combined) and poorer OS in HER2-negative patients ((B); median survival 40.7 versus 28.8 months for patients with high versus low expression respectively).

## Slide 7
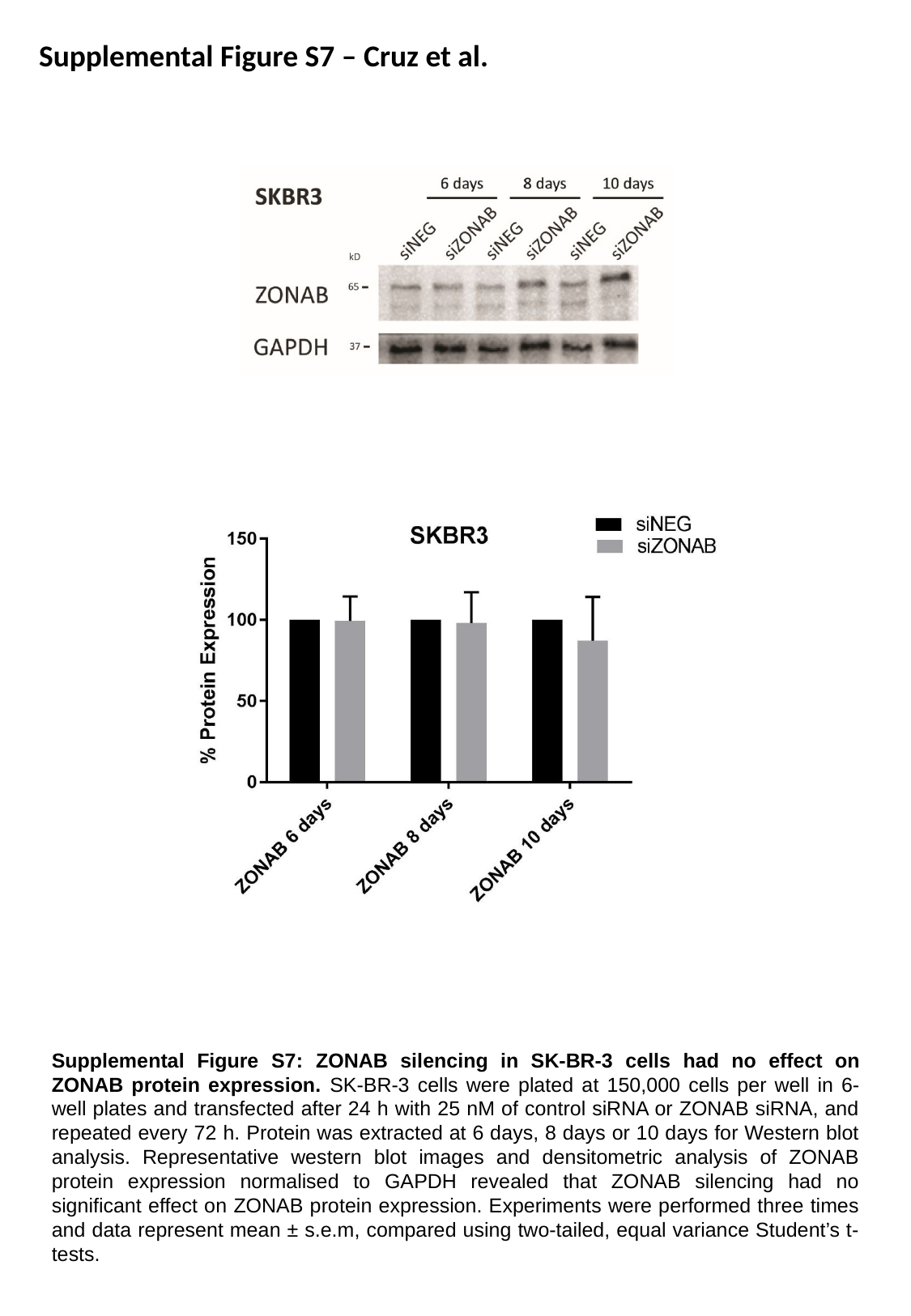

Supplemental Figure S7 – Cruz et al.
Supplemental Figure S7: ZONAB silencing in SK-BR-3 cells had no effect on ZONAB protein expression. SK-BR-3 cells were plated at 150,000 cells per well in 6- well plates and transfected after 24 h with 25 nM of control siRNA or ZONAB siRNA, and repeated every 72 h. Protein was extracted at 6 days, 8 days or 10 days for Western blot analysis. Representative western blot images and densitometric analysis of ZONAB protein expression normalised to GAPDH revealed that ZONAB silencing had no significant effect on ZONAB protein expression. Experiments were performed three times and data represent mean ± s.e.m, compared using two-tailed, equal variance Student’s t-tests.

## Slide 8
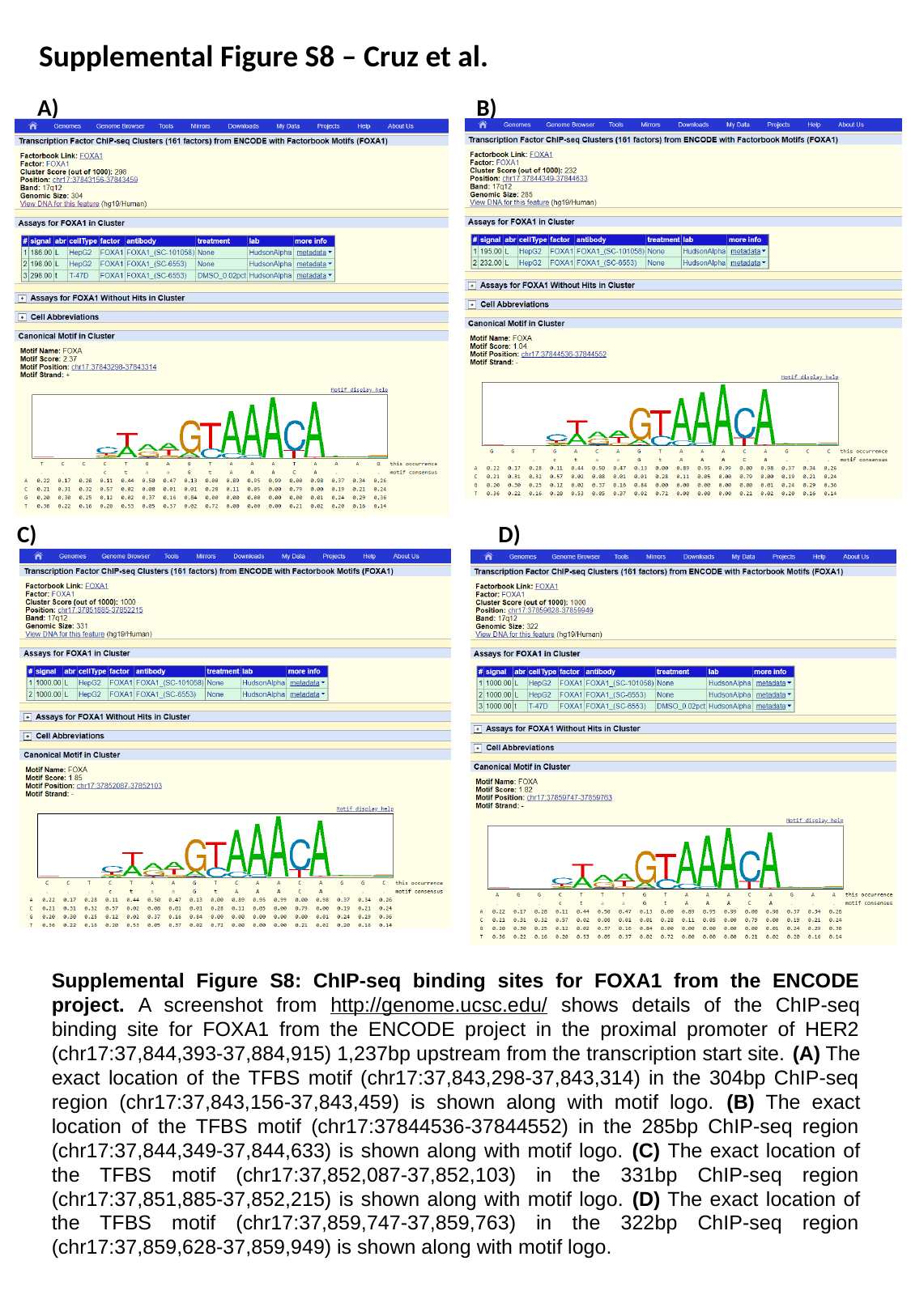

Supplemental Figure S8 – Cruz et al.
A)
B)
C)
D)
Supplemental Figure S8: ChIP-seq binding sites for FOXA1 from the ENCODE project. A screenshot from http://genome.ucsc.edu/ shows details of the ChIP-seq binding site for FOXA1 from the ENCODE project in the proximal promoter of HER2 (chr17:37,844,393-37,884,915) 1,237bp upstream from the transcription start site. (A) The exact location of the TFBS motif (chr17:37,843,298-37,843,314) in the 304bp ChIP-seq region (chr17:37,843,156-37,843,459) is shown along with motif logo. (B) The exact location of the TFBS motif (chr17:37844536-37844552) in the 285bp ChIP-seq region (chr17:37,844,349-37,844,633) is shown along with motif logo. (C) The exact location of the TFBS motif (chr17:37,852,087-37,852,103) in the 331bp ChIP-seq region (chr17:37,851,885-37,852,215) is shown along with motif logo. (D) The exact location of the TFBS motif (chr17:37,859,747-37,859,763) in the 322bp ChIP-seq region (chr17:37,859,628-37,859,949) is shown along with motif logo.

## Slide 9
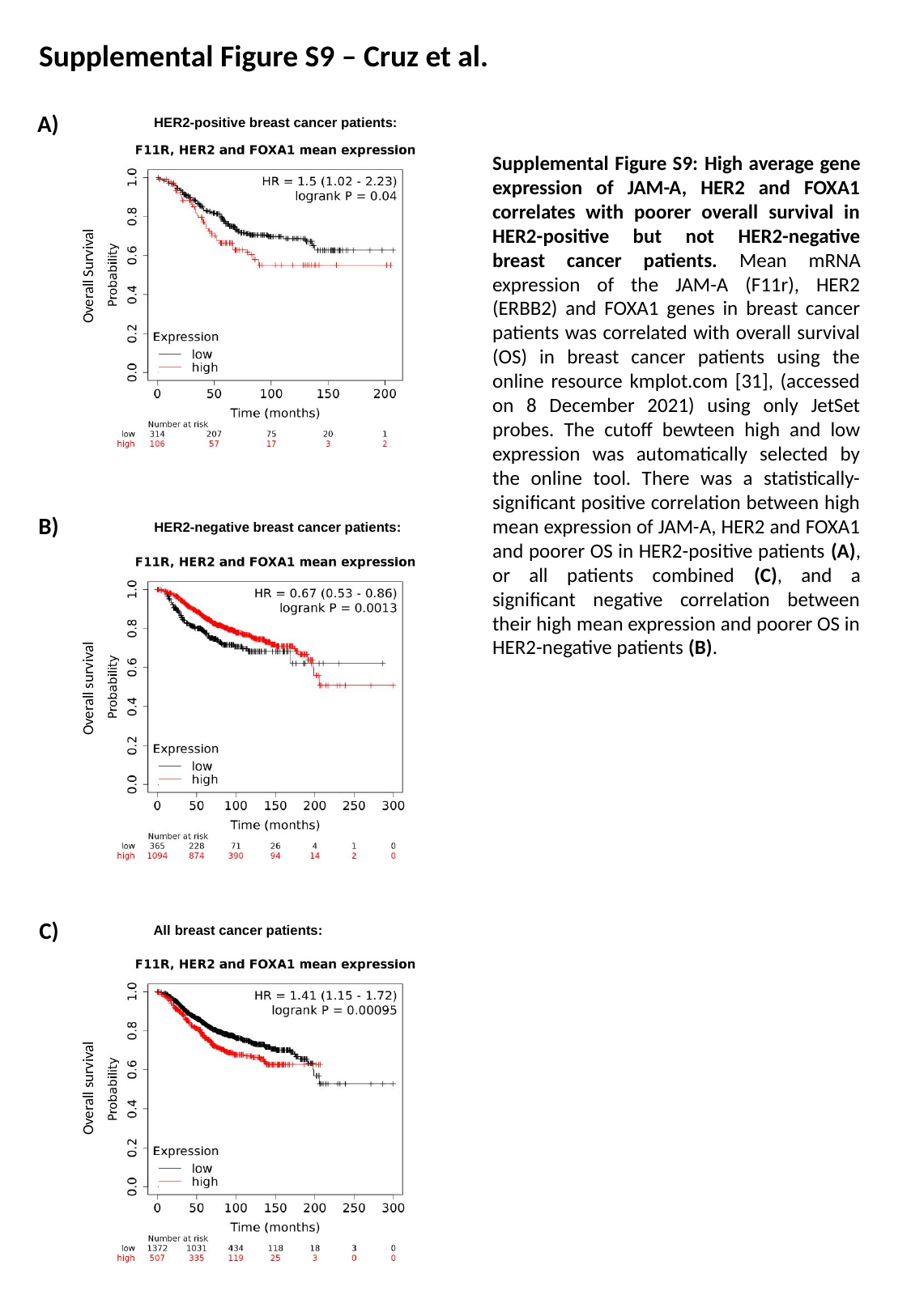

Supplemental Figure S9 – Cruz et al.
A)
HER2-positive breast cancer patients:
Supplemental Figure S9: High average gene expression of JAM-A, HER2 and FOXA1 correlates with poorer overall survival in HER2-positive but not HER2-negative breast cancer patients. Mean mRNA expression of the JAM-A (F11r), HER2 (ERBB2) and FOXA1 genes in breast cancer patients was correlated with overall survival (OS) in breast cancer patients using the online resource kmplot.com [31], (accessed on 8 December 2021) using only JetSet probes. The cutoff bewteen high and low expression was automatically selected by the online tool. There was a statistically-significant positive correlation between high mean expression of JAM-A, HER2 and FOXA1 and poorer OS in HER2-positive patients (A), or all patients combined (C), and a significant negative correlation between their high mean expression and poorer OS in HER2-negative patients (B).
Overall Survival
B)
HER2-negative breast cancer patients:
Overall survival
C)
All breast cancer patients:
Overall survival
