# Supplementary material for: A Transcriptional Link between HER2, JAM-A and FOXA1 in Breast Cancer
Source: Cells. 2022 Feb 19;11(4):735. doi: 10.3390/cells11040735 (PMC8870165; doi:10.3390/cells11040735)
Supplement: Supplementary file 1 [file cells-11-00735-s001.zip › Supplementary Tables_proofs.pdf]

Supplemental Table S1: Breast cancer datasets included in www.kmplot.com survival analysis.

Dataset name	# of patients with available clinical data
E-MTAB-365	537
E-TABM-43	37
GSE11121	200
GSE12093	136
GSE12276	204
GSE1456	159
GSE16391	55
GSE16446	120
GSE16716	47
GSE17705	196
GSE17907	54
GSE18728	61
GSE19615	115
GSE20194	45
GSE20271	96
GSE2034	286
GSE20685	327
GSE20711	90
GSE21653	240
GSE22093	68
GSE25066	507
GSE2603	99
GSE26971	276
GSE29044	79
GSE2990	102
GSE31448	71
GSE31519	67
GSE32646	115
GSE3494	251
GSE36771	107
GSE37946	41
GSE41998	279
GSE42568	121
GSE43358	57
GSE43365	111
GSE45255	139
GSE4611	153
GSE46184	74
GSE48390	81
GSE50948	156
GSE5327	58
GSE58812	107
GSE61304	62
GSE65194	164
GSE6532	82
GSE69031	130
GSE7390	198
GSE76275	265
GSE78958	424
GSE9195	77

Supplemental Table S2: Gastric cancer datasets included in www.kmplot.com survival analysis.

Dataset name	# of patients with available clinical data
GSE14210	145
GSE15459	200
GSE22377	43
GSE29272	268
GSE51105	94
GSE62254	300

Supplemental Table S3: High mean mRNA expression of JAM-A, b-catenin, FOXA1 and HER2 correlates with poorer overall survival in HER2-positive but not HER2-negative breast cancer patients.

Mean combined mRNA expression of the JAM-A (F11R), β-catenin (CTNNB1), FOXA1 (HNF3a) and HER2 (ERBB2) genes in breast cancer patients was correlated with overall survival (OS) using the online resource kmplot.com; accessed on 10 February 2022), using only JetSet probes. High versus low gene expression categories were based on a split by upper quartile expression levels; and patient survival time was also split by upper quartile levels. There was a significant positive correlation between high mean expression of all four genes and poorer OS in HER2-positive patients (*p<0.05) and all patients (***p<0.001), but not in HER2-negative patients (p=0.49).

	**Upper quartile patient survival (months):**		
**Patient category:**	**High expression** ***(patient #)***	**Low expression** ***(patient #)***	***p*** **value**
All patients	115 *(n=1,409)*	59.88 *(n=470)*	$9x10^{-5}$ (***)
HER2-positive	66 *(n=315)*	42.6 *(n=105)*	0.04 (*)
HER2-negative	115 *(n=1,094)*	106.8 *(n=365)*	0.49
